# Author Correction: Coaxially printed magnetic mechanical electrical hybrid structures with actuation and sensing functionalities

**DOI:** 10.1038/s41467-026-78963-2

**Published:** 2026-10-22

**Authors:** Yuanxi Zhang, Chengfeng Pan, Pengfei Liu, Lelun Peng, Zhouming Liu, Yuanyuan Li, Qingyuan Wang, Tong Wu, Zhe Li, Carmel Majidi, Lelun Jiang

**Affiliations:** 1 Guangdong Provincial Key Laboratory of Sensor Technology and Biomedical Instrument, School of Biomedical Engineering, Shenzhen Campus of Sun Yat-sen University, Shenzhen, 518107 PR China; 2 The State Key Laboratory of Fluid Power and Mechatronic Systems, College of Mechanical Engineering, Zhejiang University, Hangzhou, Zhejiang 310027 China; 3 Soft Machines Lab, Mechanical Engineering, Carnegie Mellon University, Pittsburgh, PA 15213 USA

Correction to: *Nature Communications* https://doi.org/10.1038/s41467-023-40109-z, published online 22 July 2023

In the version of the article initially published, in Fig. 7e, the labels “28 Hz” and “*B*_act_ = 150 mT” should have read “26 Hz” and “*B*_act_ = 165 mT”, respectively. Additionally, in the Source Data file, the data for Supplementary Fig. 22a2 were incorrect and have now been replaced. The Supplementary information has also been updated to include the correct Supplementary Fig. 22a2. The correction does not affect the conclusions of the article. This correction has been made to the HTML and PDF versions of the article. For comparison, the original Fig. 7 and Supplementary Fig. 22 are available as Supplementary Information accompanying this amendment.

## Supplementary information

Original, uncorrected Fig. 7 and Supplementary Fig. 22

