# Supplementary material for: Coaxially printed magnetic mechanical electrical hybrid structures with actuation and sensing functionalities
Source: Nat Commun. 2023 Jul 22;14:4428. doi: 10.1038/s41467-023-40109-z (PMC10363174; doi:10.1038/s41467-023-40109-z)
Supplement: Supplementary file 1 — Supplementary Information [file 41467_2023_40109_MOESM1_ESM.pdf]

Supplementary Information for

Coaxially Printed Magnetic Mechanical Electrical Hybrid Structures with Actuation and Sensing Functionalities

Yuanxi Zhang[1,#], Chengfeng Pan[2,#], Pengfei Liu[1,#], Lelun Peng[1], Zhouming Liu[1],Yuan Yuan Li[1], Qingyuan Wang[1], Tong Wu[1], Zhe Li[1*], Carmel Majidi[3*], and Lelun Jiang[1*]

[1]Guangdong Provincial Key Laboratory of Sensor Technology and Biomedical Instrument, School of Biomedical Engineering, Shenzhen Campus of Sun Yat-sen University, Shenzhen 518107, PR China

[2]The State Key Laboratory of Fluid Power and Mechatronic Systems, College of Mechanical Engineering, Zhejiang University, Hangzhou, Zhejiang 310027, P.R. China

[3]Soft Machines Lab, Mechanical Engineering, Carnegie Mellon University, Pittsburgh, PA 15213, USA

[#]These authors contributed equally to this work

[*]Correspondence: lizhe28@mail.sysu.edu.cn (Z.L.), cmajidi@andrew.cmu.edu (C.M.), jianglel@mail.sysu.edu.cn (L.J.)

The PDF file includes

Supplementary Tables 1-3

Supplementary Figures 1-48

Supplementary Table 1. Strategies reported in the literature for integrating a flexible matrix with conductive materials

**Summary**	**Fabrication method**	**Materials**	**Structural complexity**	**References**
Multiple separated steps	Embedding	Liquid metal microdroplets + NdFeB PDMS	Intermediate (Patterns)	*Sci. Adv. 2021* [1]
	Thin-film coating	Liquid metal/ Cu/Au + NdFeB @ PDMS film	Low (Single-layer pattern)	*Adv. Funct. Mater. 2021* [2]<br>*Adv. Mate 2019 2020* [3-4]
	Assembly	Coils (Cu) + NdFeB @ PDMS film	Intermediate (Multilayers)	*Adv. Funct. Mater.2020, 2022* [5-6]

Supplementary Table 2. The MME fiber in this study in comparison with representative core-sheath fiber structures with a liquid metal core reported in the literature

Different core-sheath structure			Fabrication method	Functional components			Enabled hybrid functions			Typical applications	Ref.
Design	Core material	Sheath material		Mechanical	Electrical	Magnetic	Programming magnetization	Somatosensory actuation	Hybrid actuation & energy transfer		
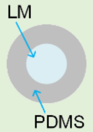 	Liquid metal (LM)	PU<br>PDMS	3D shape programming + di-coating	√	√	×	×	×	⍻<br>**(only wired energy transfer)**	● Mechanical sensor<br>● Flexible circuit	7-8
		PU<br>PVDF-HFP-TFE	Coaxial wet-spinning	√	√	×	×	×	⍻<br>**(only wired energy transfer)**	● Pressure sensor<br>● Triboelectric<br>● Joule heating	9-11
		SEBS<br>PDMS	Coaxial printing	√	√	×	×	×	⍻<br>**(both wired and wireless energy transfer)**	● Mechanical sensor<br>● Pressure sensor<br>● Wireless energy transfer	12-15
		Silicone<br>Ecoflex<br>SEBS	Template molding + injecting	√	√	×	×	×	⍻<br>**(only wired energy transfer)**	● Metamaterial<br>● Stretchable antennas<br>● Triboelectric<br>● Contactless sensing<br>● Phase transition	16-22
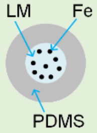 	Iron particles & liquid metal	Ecoflex<br>Latex		√	√	√	×	×	⍻<br>**(only wired energy transfer)**	● Actuator<br>● Variable stiffness	23-24
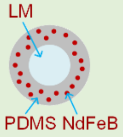 	**Liquid metal**	**Magnetic particles (NdFeB) & PDMS**	**Coaxial printing**	√	√	√	√	√	√	● Mechanical sensor<br>● Triboelectric<br>● Wireless energy transfer<br>● Actuator<br>● Somatosensory actuation	**Our work**

Supplementary Table 3. Comparison of this work with the manipulators/actuators with synchronous actuation and deformation sensing reported in the literature

**Structural**	**Materials & components**	**Stimuli-responsive**	**Response**	**Reference**
Integration	Conductive photothermal hydrogel	Heat	Slow & Wireless	*Sci. Robot.* 2021 [25]
Integration	Graphite @ PVDF film	Light	Slow & Wired	*Adv. Mater.* 2020 [26]
Assembly	TENG sensor & Soft gripper	Pneumatic	Fast & Wired	*Nat. Commun.* 2020 [27]
Assembly	Resistance strain sensor & Soft gripper	Pneumatic	Fast & Wired	*Adv. Mater.* 2018 [28]
Assembly	Optical sensor & Soft actuator	Pneumatic	Fast & Wired	*Sci. Robot.* 2016 [29]
**Integration**	**NdFeB @ PDMS + Liquid metal**	**Magnetic field**	**Fast & Wireless**	**This work**

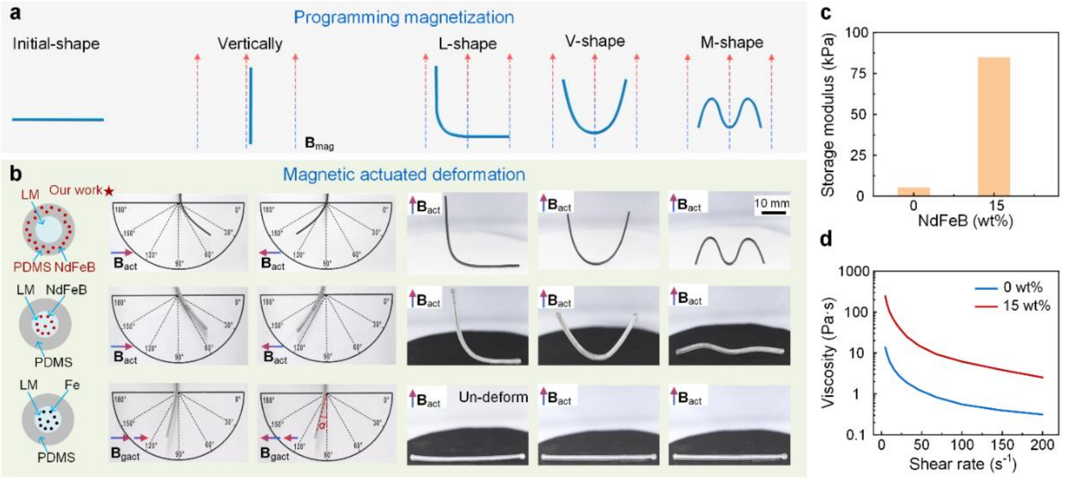

Supplementary Figure 1. Comparison between the MME fiber in this work and existing core-sheath fibers (sheath materials: PDMS or NdFeB @ PDMS; core materials: liquid metal, Fe @ liquid metal or NdFeB @ liquid metal). (a) Schematic illustration of the magnetization process for fibers deformed into different geometries. (b) Experimental results showing magnetically driven deformation of fibers prepared with different materials of different magnetization profiles. After programmed magnetization (L-shape, V-shape, M-shape), our MME fibers can be deformed into the pre-designed shape under magnetic actuation $\mathbf{B}_{act}$. The NdFeB @ liquid metal ferrofluid-based fiber can only be magnetically actuated into simple shapes (cannot be deformed into a complex M-shape). The Fe @ liquid metal ferrofluid-based fiber can only be bended/deformed in a gradient magnetic field $\mathbf{B}_{gact}$, but cannot be deformed into complex shapes. (c) Storage modulus and viscosity of liquid metals with different NdFeB contents. (d) The viscosity of liquid metals with different NdFeB contents. The B_{act} = 18 mT, $\nabla|\mathbf{B}_{act}|$ = 0 mT/mm, and the B_{gact} = 90 mT, $\nabla|\mathbf{B}_{gact}|$ = 4 mT/mm.

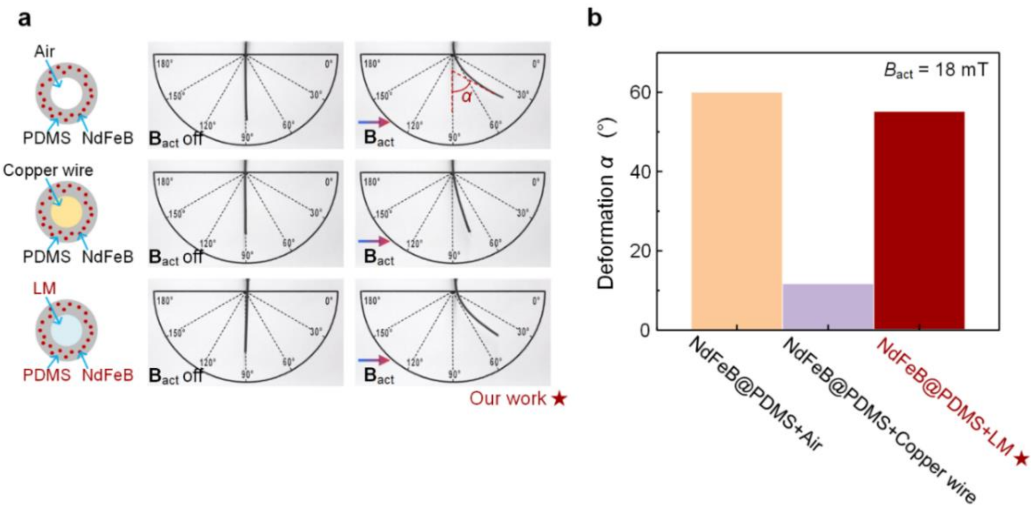

Supplementary Figure 2. Comparison of deformation capabilities and flexibility of different core-sheath fibers under magnetic actuation. (a) Deformation of different core-sheath fibers under the same actuation magnetic field (B_{act} =18 mT; core materials: air, copper wire, or liquid metal). (b) Deformation angles for different core-sheath fibers. Unlike the high-modulus copper wire that constrains the deformation, the liquid metal core with high fluidity does not evidently impair the magnetically induced deformation. The MME fiber thus has similar flexibility as its hollow counterpart (filled with air).

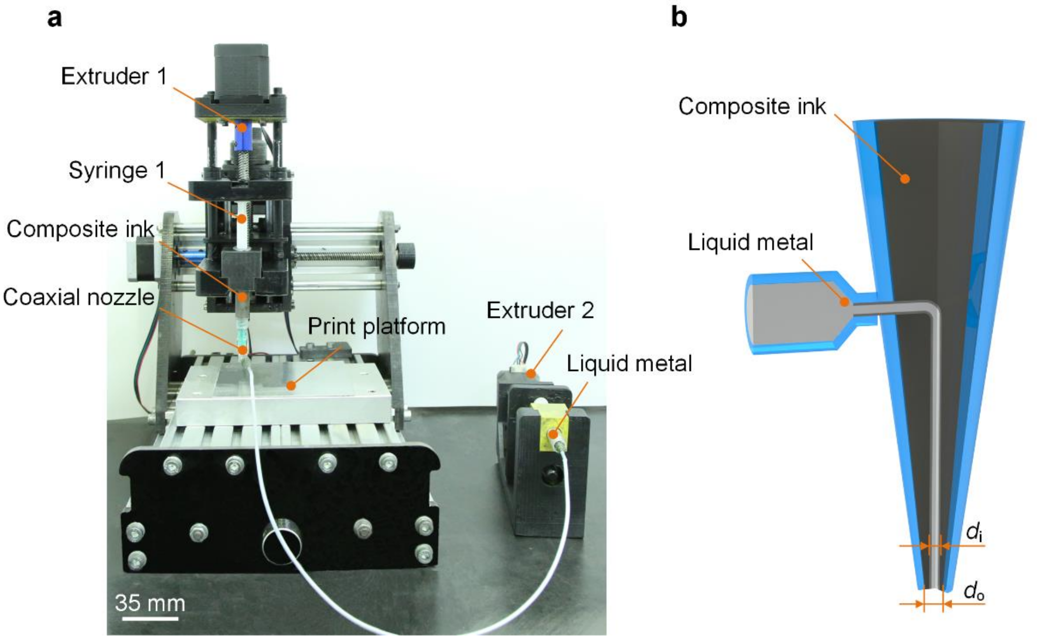

Supplementary Figure 3. Experimental setup for coaxial printing. (a) The custom-designed coaxial printer. (b) Schematic diagram of the coaxial nozzle; the inner nozzle diameter d_i is 380 μm, and the nozzle diameter d_o is 800 μm. The coaxial printer is based on a cartesian gantry system (AGS1000, Aerotech). It is consisted of three step motors (17HS4401, Usongshine, China), extruder 1 and extruder2, a syringe (MJZSQ-2.5, Guangzhou Meijun Biological Technology Co., Ltd, China), and a print platform. The X/Y/Z step motors would control the motion of the syringe, achieving numerically controlled 3D motion of the coaxial nozzle based on G-codes. The syringes' composite ink and liquid metal are simultaneously extruded from the coaxial nozzle.

The remanence increases with the increase of NdFeB content; a high content of NdFeB particles can help enhance the magnetically actuated deformability of the MME fiber/structure. However, with the increase of NdFeB contents, yield stress of the composite ink also increases, making it hard to print the composite ink; also a high NdFeB content also reduces the flexibility of the MME fiber/structure. As shown in Supplementary Fig. 4c and d, as the NdFeB content increases, elastic modulus of printed MME fibers increases and the tensile strength decreases. After systematic investigation, the composite ink was optimized to have a NdFeB particle content of 50 wt% (the mass ratio of PDMS to NdFeB particles is 1:1).

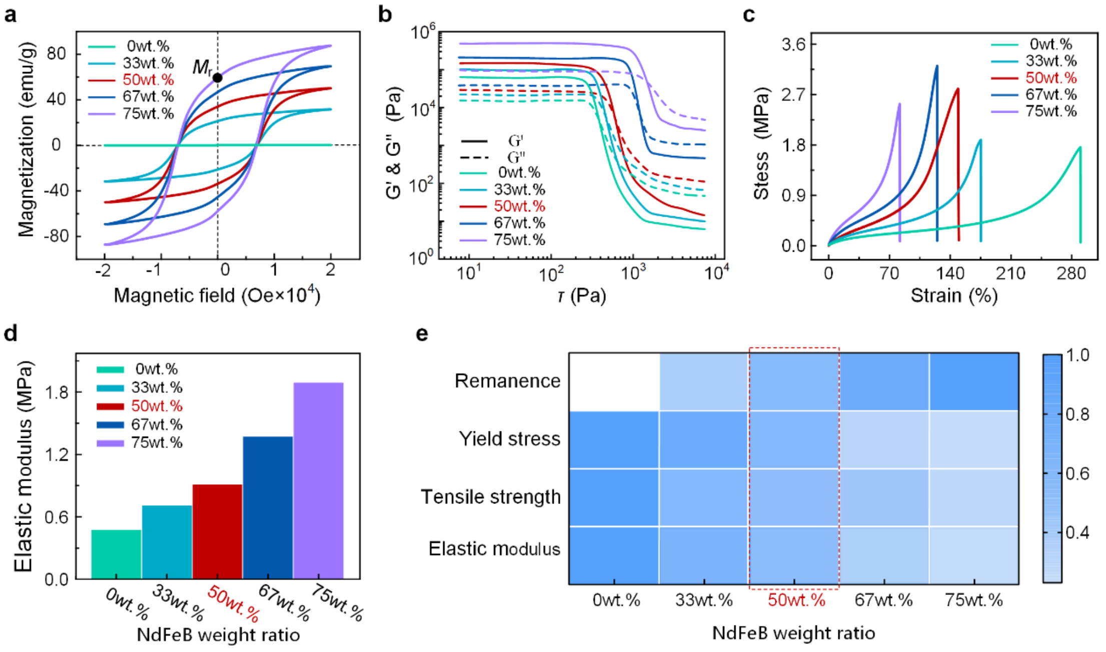

Supplementary Figure 4. Properties of composite inks with different NdFeB weight ratios. (a) Magnetization hysteresis loops for composite inks with different NdFeB weight ratios. (b) The shear storage modulus G' and shear loss modulus G" for different composite inks. (c) The tensile stress-strain curves for fibers printed with composite inks of different NdFeB weight ratios (stretching rate: 1 mm/s). (d) Elastic modulus of fibers printed with composite inks of different NdFeB weight ratios. (e) Heatmap summarizing the properties (remanence, yield stress, tensile strength, and elastic modulus) of composite inks with different weight ratios of NdFeB.

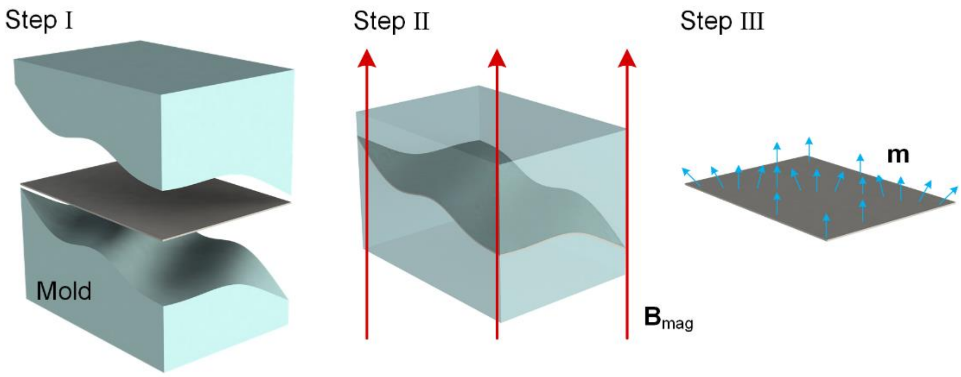

Supplementary Figure 5. Schematic diagram of the magnetization process. In step I, the cured unmagnetized MME structure was placed in a mold with a predesigned geometry. In step II, the unmagnetized MME structure was elastically deformed by the mold and placed in a pulsed magnetic field $\mathbf{B}_{mag}$ (about 3 T). Under the action of a pulsed magnetic field $\mathbf{B}_{mag}$, the MME structure would reache its saturation magnetization, with the magnetization direction same as the pulsed magnetic field $\mathbf{B}_{mag}$. In step III, after the MME structure is released from the mold and re-coiled to its initial deformation free geometry, a magnetization profile **m** would be imparted onto the MME structure. In this process, magnetization profile **m** of the MME structure can be controlled by designing the geometry of the mold and adjusting the pulsed magnetic field $\mathbf{B}_{mag}$.

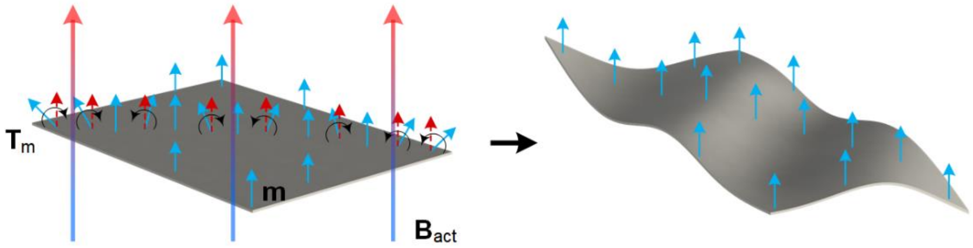

Supplementary Figure 6. Schematic diagram of the magnetically actuated deformation of a magnetized MME structure. Interaction between the external actuation magnetic field $\mathbf{B}_{act}$ and the magnetization profile **m** will generate a spatially varying magnetic torque $\mathbf{T}_m$, across the MME structure. $\mathbf{T}_m$ would deform the MME structure till an equilibrium state is achieved. The direction of the magnetization profile **m** tends to coincide with the direction of the actuation magnetic field $\mathbf{B}_{act}$.

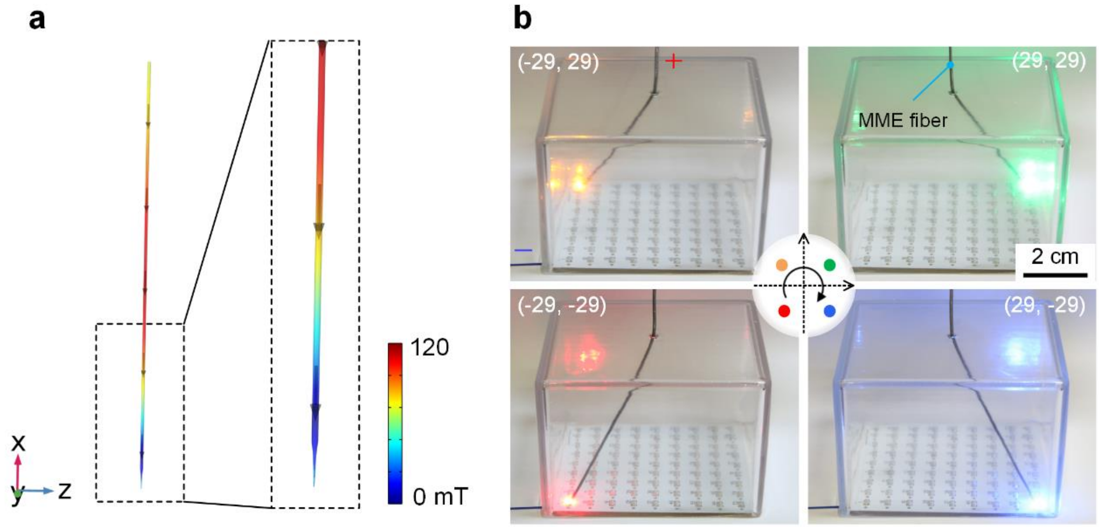

Supplementary Figure 7. Magnetic actuation of the 1D MME fiber. (a) Simulation result of magnetization profile of 1D MME fiber. (b) Controlled by an external actuation magnetic field, the 1D MME fiber can be precisely actuated to electrically connect and light LED pixels (the distance between LED pixels is 6.5 mm) in a confined space.

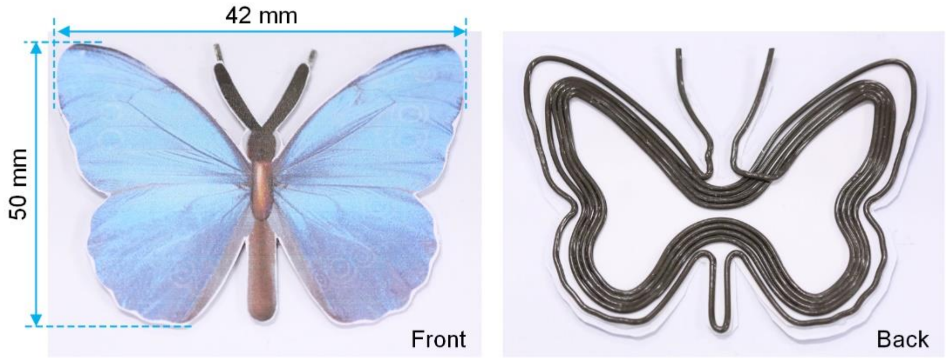

Supplementary Figure 8. The coaxially printed butterfly robot with a 2D MME coil structure as the skeleton (size: 42 × 50 × 0.8 mm^3).

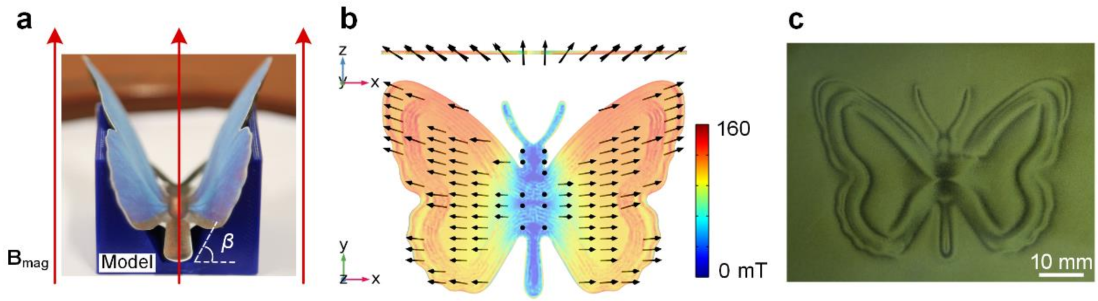

Supplementary Figure 9. Magnetization of the butterfly robot. (a) The magnetization process of the butterfly robot. Assisted by a magnetization mold, the butterfly robot is deformed to a flying gesture with a deformation angle β of 70°. (b-c) The magnetization profile **m** of the magnetized butterfly robot; (b) simulation result, (c) experimental result. When the magnetic development card is close to the butterfly robot, the iron powder inside it is affected by the magnetic force of the butterfly robot, and gathers in the area with higher magnetic field strength in the z direction, causing the color of the magnetic development card to change from brown to black.

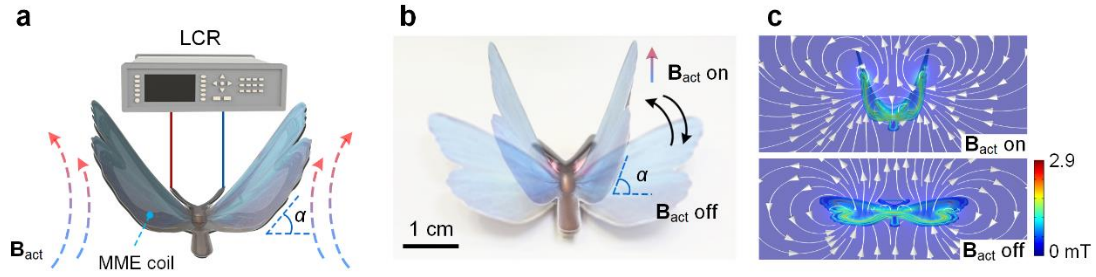

Supplementary Figure 10. Hybrid actuation and sensing capabilities for a butterfly robot. (a) The inductance of the 2D MME coil during the deformation of the butterfly robot is measured by the inductance, capacitance, resistance (LCR) meter. (b) Controlled by the actuation magnetic field $\mathbf{B}_{act}$, the butterfly robot can be deformed to a flying gesture by the magnetic force. (c) Simulation results of the induced magnetic field $\mathbf{B}_{ind}$ caused by the deformation of the butterfly robot. At a larger deformation angle α, the inductive magnetic field $\mathbf{B}_{ind}$ generated by two halves of the deformed coil will gradually repel and cancel each other.

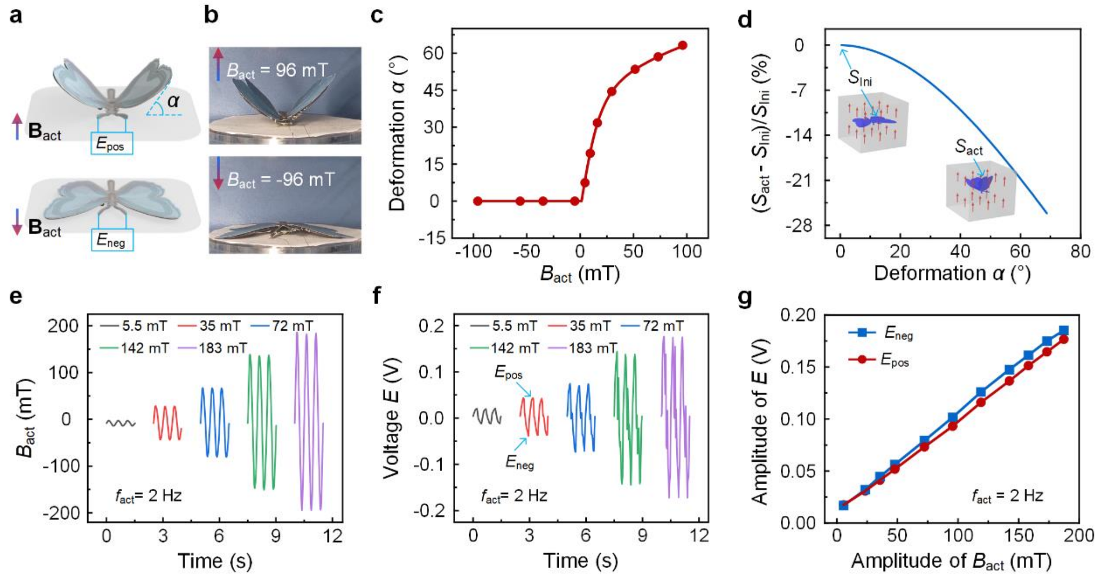

Supplementary Figure 11. Power generation performance of the butterfly robot driven by a low frequency magnetic field. (a) Measurement of the induced voltage generated by the butterfly robot in response to a low-frequency magnetic field. (b) Deformation of the butterfly robot driven by a low-frequency magnetic field. When a downward the magnetic field is applied, the substrate would limit the deformation of the butterfly robot. (c) Deformation angle of the butterfly robot as a function of the actuation magnetic field strength. (d) The equivalent area *S* of the butterfly robot in the direction of the magnetic field (or the vertical direction in this test) varies with the deformation angle α. As the deformation angle α increases, the equivalent area decreases gradually. (e) A low-frequency magnetic field with increasing strength (f_{act} = 2 Hz) for butterfly actuation. (f) Output voltage of the butterfly robot as a function of the actuation magnetic field strength (5.5-187 mT). The output voltage increases with the increase of the magnetic field strength. (g) Dependence of the output voltage *E* on the magnetic field strength B_{act}.

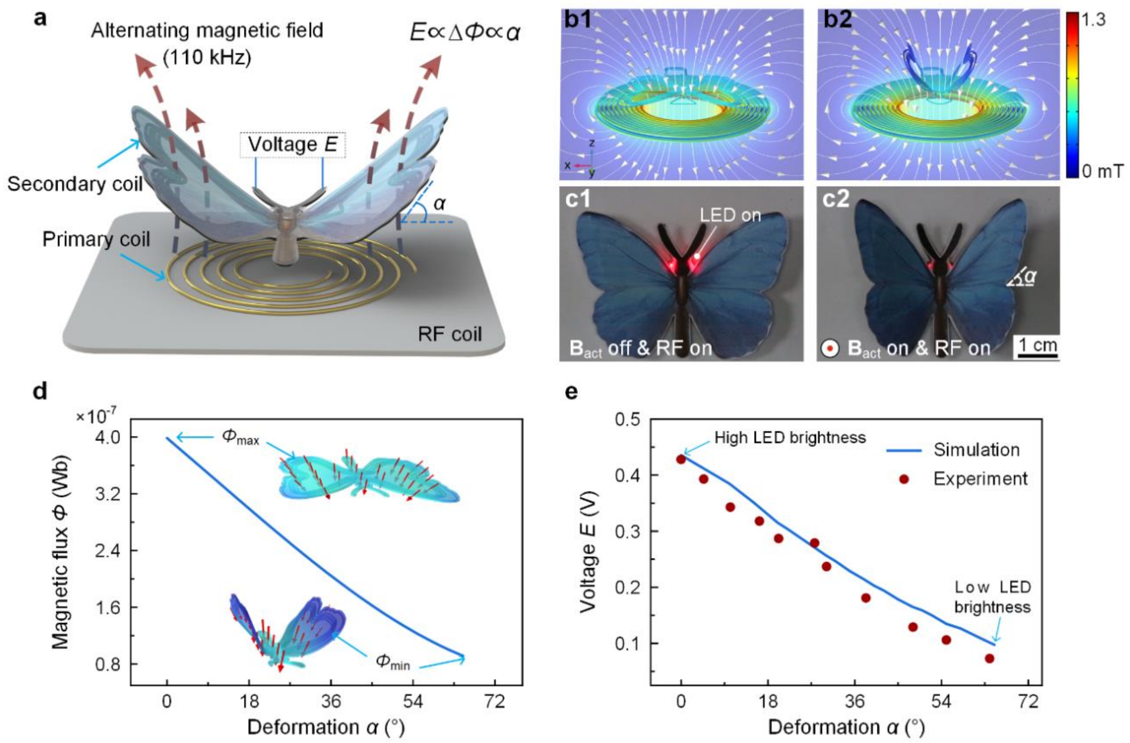

Supplementary Figure 12. Performance of the radio frequency (RF) wireless energy transmission of the butterfly robot. (a) Schematic illustration of the RF wireless energy transmission. The printed 2D MME coil skeleton served as the secondary coil in the butterfly robot for wireless power transmission. (b) Magnetic field distribution of the butterfly robot at different bending angles in the RF wireless energy transmission. (c) Along with the deformation of the butterfly robot (driven by a low-frequency actuation magnetic field B_{act}), LEDs on the butterfly robot can also be lit by another high-frequency magnetic field generated by the RF coil. As the deformation angle α increases, the induced voltage would decrease, and brightness of the LED light decreases correspondingly. (d) The magnetic flux variation of the butterfly robot at different bending angle α. As the bending angle α of the butterfly robot increases, the magnetic flux decreases gradually. (e) Simulation and experimental results of the induced voltage of the secondary coil under different bending angle α. The induced voltage of the secondary coil would decrease with the increase of the bending angle α.

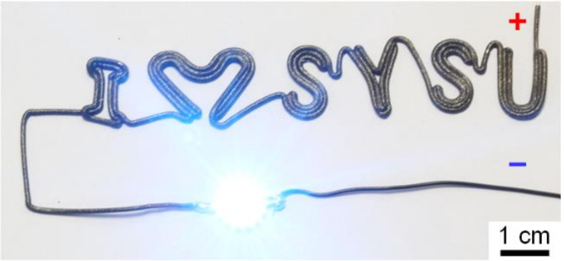

Supplementary Figure 13. Coaxially printed MME letters with complex geometries (I ♥ SYSU); the conductive core of the MME structure can be used to light a LED.

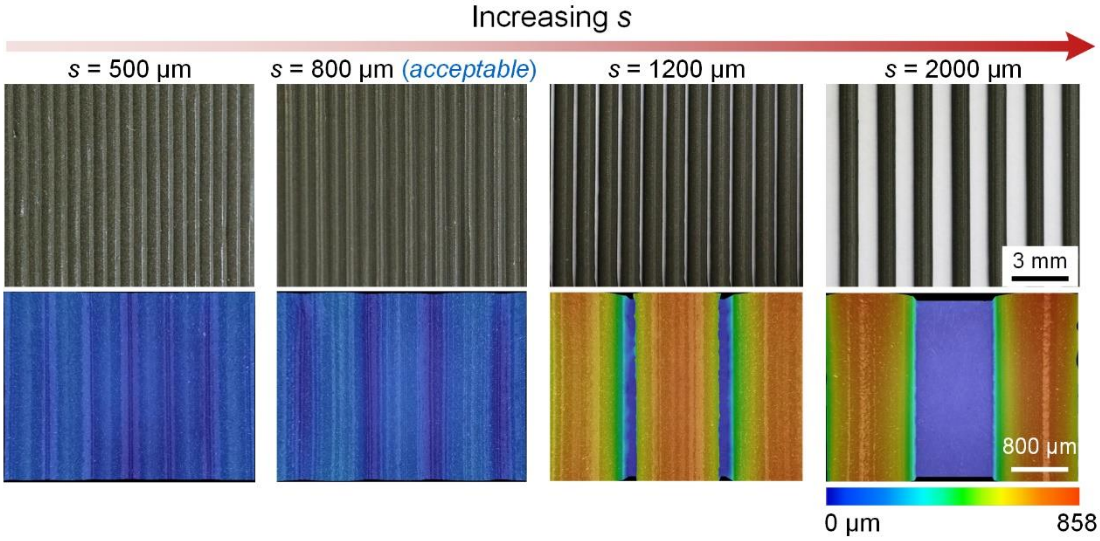

Supplementary Figure 14. Optical images and 3D morphology (measured by the ultra-depth three-dimensional microscope) of MME fibers printed with different fiber spacing *s* (outer diameter of the fiber d_m: about 830 μm). As *s* increases, overlapping between adjacent fibers decreases. At *s* = 1200 μm or 2000 μm, adjacently printed MME fibers were completely separated from each other. At *s* = 800 μm (slightly smaller than d_m), adjacent MME fibers were sufficiently bonded together without impairing the electrical conductivity of the liquid metal core.

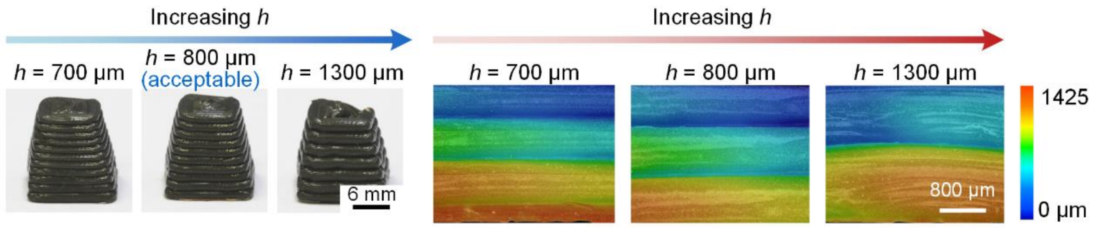

Supplementary Figure 15. Morphology of 3D MME structures printed with different layer spacing *h*. As *h* increases, overlapping between adjacent layers decreases, affecting the structure fidelity; meanwhile, a smaller h would affect MME structures' conductivity. At *h* = 800 μm, the 3D MME structure with high structure fidelity and electrical conductivity can be fabricated.

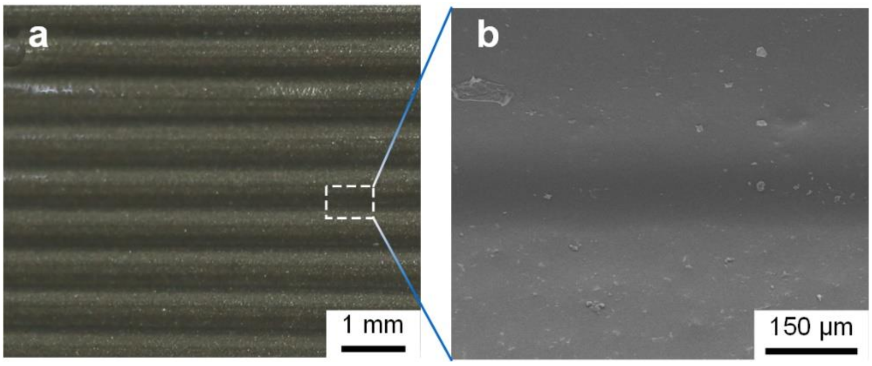

Supplementary Figure 16. Morphology of the MME fibers. (a) An optical image showing parallelly printed MME fibers (outer diameter of the fiber d_m: about 830 μm; spacing *s:* 800 μm. (b) The SEM image showing the bonding area between two adjacent MME fibers; as shown in (b), under optimized printing parameters, adjacent MME fibers were intimately bonded together.

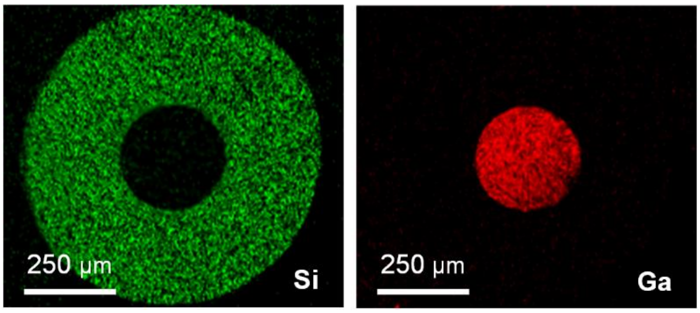

Supplementary Figure 17. Images showing elemental distribution at the cross-section of a typical MME fiber (outer diameter d_m: about 830 μm; inner diameter for the liquid metal core d_l: about 270 μm). Ga elements (from the liquid metal) are mainly distributed in the core. Si (from the PDMS of the composite ink), elements are concentrated in the sheath

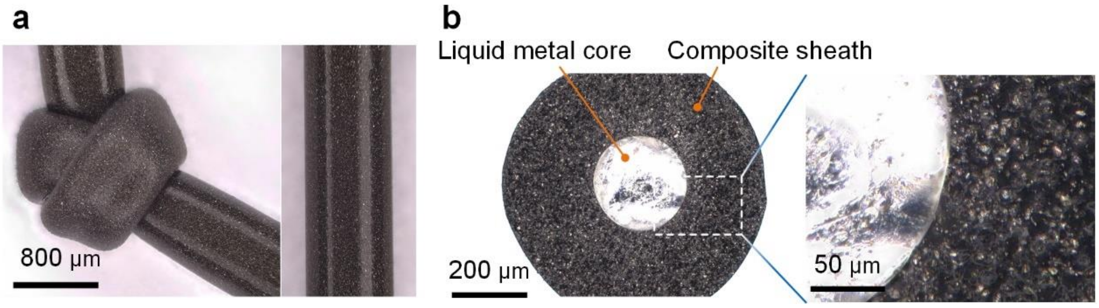

Supplementary Figure 18. Morphology of the MME fiber. (a) Optical images demonstrating the flexibility of the MME fiber (diameter: about 830 μm). (b) Cross-section image of a typical MME fiber, showing that an integral core-sheath structure.

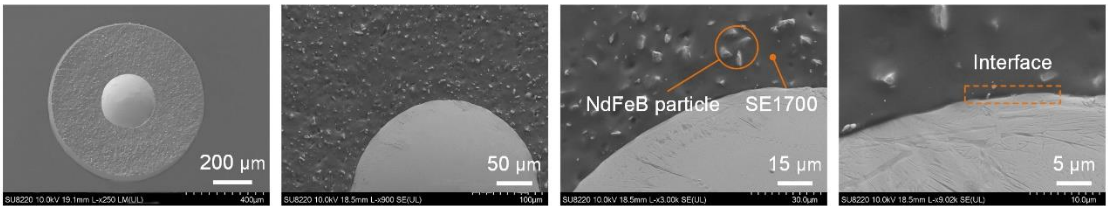

Supplementary Figure 19. High-resolution SEM images of the MME fiber cross-section showing a clear boundary between the NdFeB & PDMS composite sheath and the liquid metal core. The NdFeB particles are uniformly distributed in the composite sheath, and the liquid metal fills the cavity of the sheath.

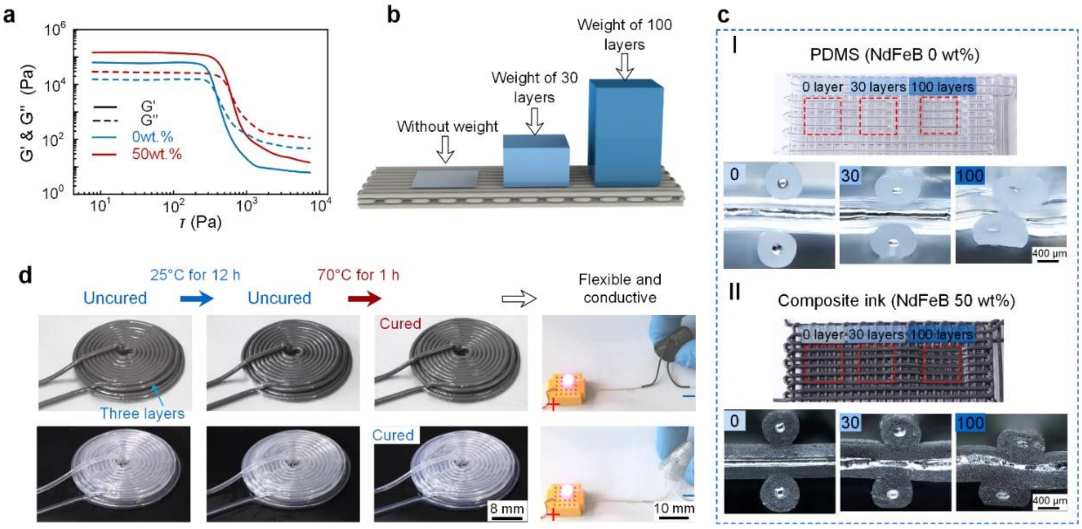

Supplementary Figure 20. Printing performance with two different sheath materials (pure PDMS*:* 0 wt% NdFeB in PDMS; the composite ink*:* 50 wt% NdFeB in PDMS). (a) The shear storage modulus G' and shear loss modulus G" of composite inks containing different weight fractions of NdFeB particles. (b) Schematic illustration of coaxially printed structures with different equivalent layer thicknesses; weight of the 30-layer structure for the pure PDMS and the composite ink sheath is 1.56 g and 1.83 g respectively; weight of the 100-layer structure for the pure PDMS and the composite ink sheath is 5.2 g and 6.1 g, respectively. (c) Deformation of the fiber at the junction. In the test mimicking a 30-layer structure, both the pure PDMS and composite ink sheath maintained good structural integrity. Under the weight of 100 layers, although deformation of the fiber at the bottom layer was observed (which obviously was owing to the exerted weight), an integral core-sheath structure was still maintained and the liquid metal core was not squeezed out to rupture the sheath. (d) Coaxially printed three-layer coil structures left at ambient environments before curing. The three-layer coil structures were printed with two different inks (pure PDMS*,* or the composite ink). Even left at the ambient environment (25 °C) for 12 hours, the uncured structure (including the connecting fibers in the dangling position at the very top layer) maintained excellent structural fidelity. After curing at 70 °C for 1 hour, the cured three-layer coil structures demonstrated good conductivity and flexibility as their counterparts that were cured immediately after printing.

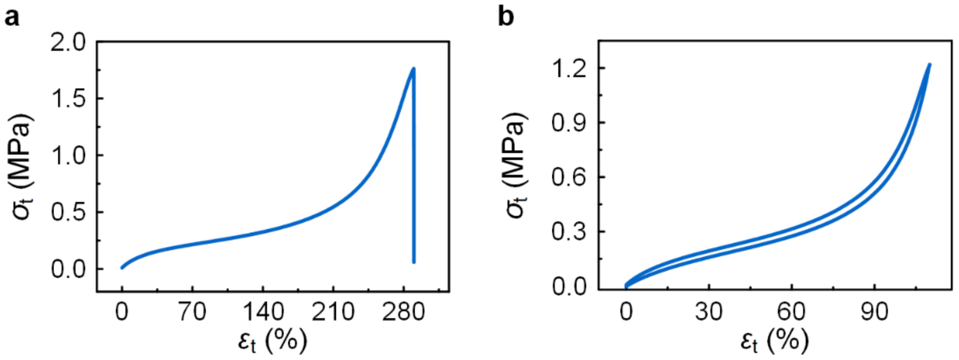

Supplementary Figure 21. Tensile properties of a PDMS fiber with core-sheath structure. (a) The tensile stress-strain curve of a PDMS fiber. (b) The 1st loading-unloading test for a MME fiber. The coaxially printed fiber was used for tensile experiments (gauge length: 25 mm; outer diameter d_m: about 830 μm; inner diameter for the liquid metal core d_l: about 270 μm), and the stretching rate is 0.5mm/s.

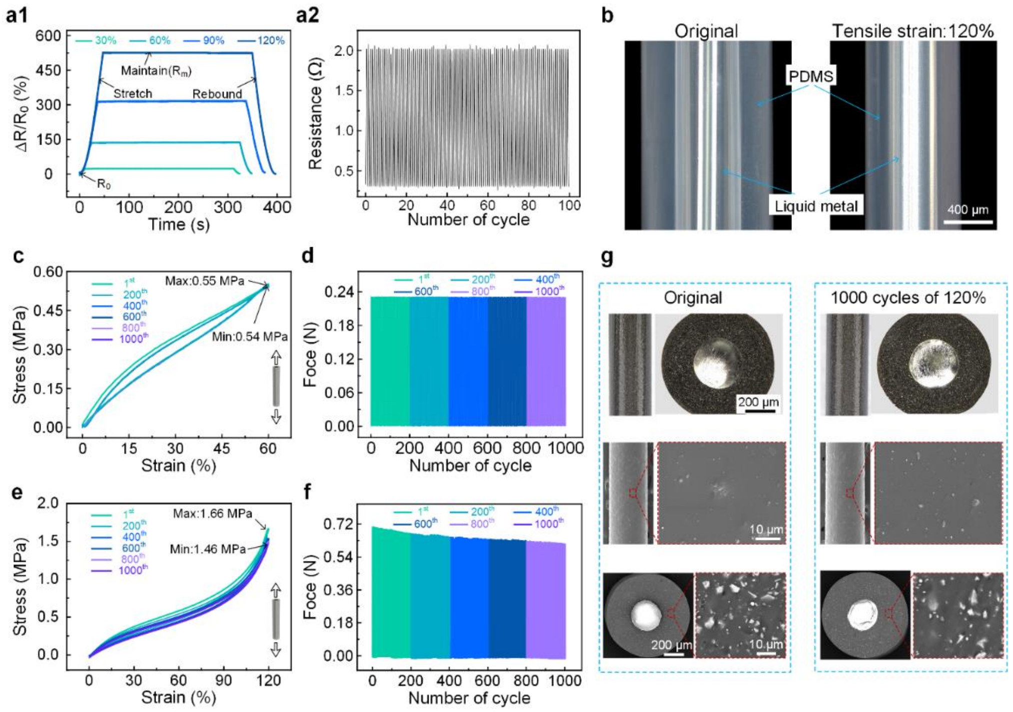

Supplementary Figure 22. Characterization of the electrical and mechanical stability and durability of the MME fiber. (a1) Electrical resistance in stretching tests of different tensile strains (fiber length: 35 mm; initial electrical resistance R_0: ~ 0.31 Ω); the MME fiber was stretched to different strains, maintained at the maximum strain for 300 seconds and then unloaded; loading and unloading rate:1mm/s. (a2) Changes in electrical resistance during the cyclic stretching (tensile strain: 120%); loading/unloading rate: 1mm/s. (b) Optical images of the pristine core-sheath fiber and the stretched fiber at 120% strain. (c) Cyclic loading-unloading tests for the MME fiber (stretching rate: 1 mm/s; tensile strain: 60%). After 1000 cycles' tensile fatigue tests, the maximum stress of the fiber was reduced from 0.55 MPa to 0.54 MPa (reduced by 1.8%). (d) Tensile force experienced by the MME fiber during the cyclic loading-unloading tests at a tensile strain of 60%. (e) Cyclic loading-unloading tests for the MME fiber (stretching rate: 1 mm/s; tensile strain: 120%). After 1000 cycles' tensile tests, the maximum stress of the fiber was reduced from 1.66 MPa to 1.46 MPa (reduced by 12%). (f) Variation of the tensile force experienced by the MME fiber during the cyclic loading-unloading tests at a tensile strain of 120%. (g) Optical and SEM images showing the morphology of the core-sheath MME fiber (before stretching and after 1000 cycles' stretching). After 1000 cycles' testing, no obvious micro-crack or particle detachment would be detected on the surface or the cross-section.

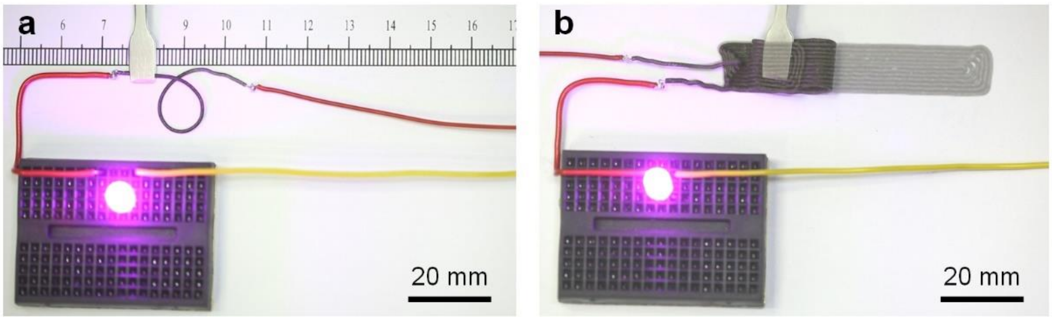

Supplementary Figure 23. Conductivity of the curved MME fiber. (a) A curved MME fiber can be used to light a LED. (b) A MME coiled in the folded state is still conductive to light the LED. Voltage: 3.0 V DC.

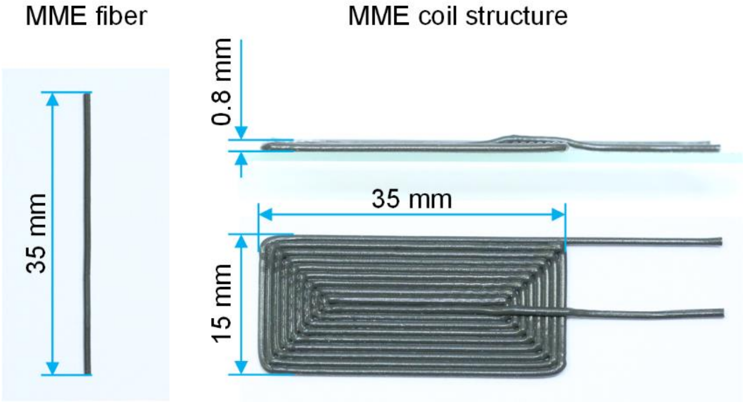

Supplementary Figure 24. A MME fiber and a MME coil structure fabricated by coaxial printing.

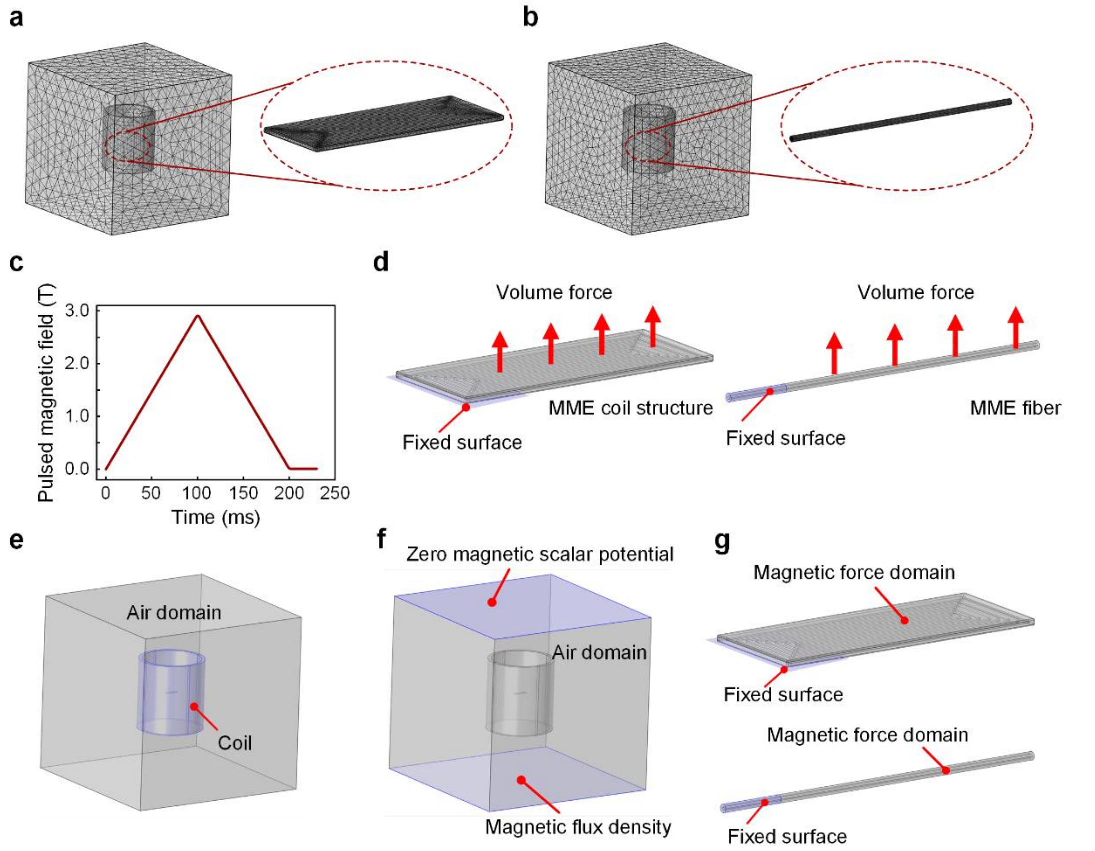

Supplementary Figure 25. The finite element analysis magnetization and magnetically actuated deformation of coaxially printed MME fiber and MME coil structures. (a) Meshed model of the MME coil structure used in the FEA. (b) Meshed model of the MME fiber structure used in the FEA. (c) The pulsed magnetic field for magnetizing. (d-e) Boundary condition settings for the solid mechanics module and the magnetic field module for the finite element models of the MME fiber and MME coil structure. (f-g) Boundary condition settings for the solid mechanics module and the magnetic field module in the finite element model of the magnetically actuated deformation process of the MME fiber and MME coil structure.

The magnetization and magnetic actuation deformation of MME structures were analyzed by COMSOL Multiphysics 6.0 (COMSOL Inc., Sweden). The multi-physics models of FEA were built in a 500×500×500 mm^3 cube space, which mainly consisted of air, coil (180 mm in diameter), and MME structure. The tetrahedral grid was used to divide the model, and the grid size ranged from 0.16 mm to 2.2 mm, as shown in Supplementary Fig. 25a-b. The FEA of magnetization includes two analysis steps: the calculation of the structure bending process under external volume force using the solid mechanics module in stationary and the analysis of the MME structure magnetization profile under 3 T pulsed magnetic field using the magnetic field module in time dependent. The boundary condition settings for the solid mechanics module and the magnetic field module for the finite element models of the MME fiber and MME coil structure as shown in Supplementary Fig. 25d. The length of the fixed surface is 9 mm, and the volume force is applied to deform the MME fiber and MME coil structure. The folded MME structure was magnetized using a pulse current to the coil, which could generate a 3 T pulse magnetic

field in the magnetism module, as shown in Supplementary Fig. 25e. The pulsed magnetic field is shown in Supplementary Fig. 25c.

After calculating the magnetization profile **m** in the MME structure, the magnetic actuation deformation was analyzed by coupling the magnetic field-no current module with the solid mechanics module (Supplementary Fig. 25f). The magnetic actuation deformation of each step was analyzed by interactive calculation of magnetic force using the magnetic field-no current module and the deformation using the solid mechanics module. The magnetic force on the MME structure was calculated by integrating the magnetic stress tensor induced by the interaction between actuation magnetic field $\mathbf{B}_{act}$ and the calculated magnetization profile **m**. The boundary condition settings of the MME fiber and MME coil structure as shown in Supplementary Fig. 25g. Finally, the final magnetic actuation deformation of the MME structure could be obtained by iterative calculation in multi-steps.

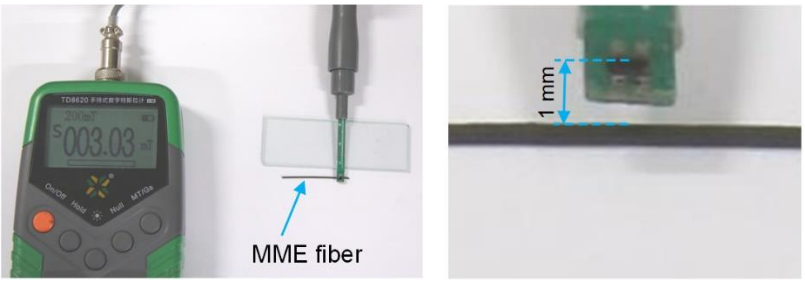

Supplementary Figure 26. Measurement of the magnetic flux density **B** at 1 mm from the MME fiber with a gauss meter.

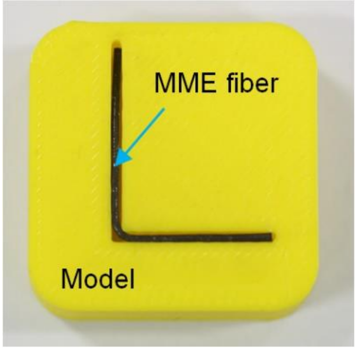

Supplementary Figure 27. The L-shaped mold for magnetizing a MME fiber.

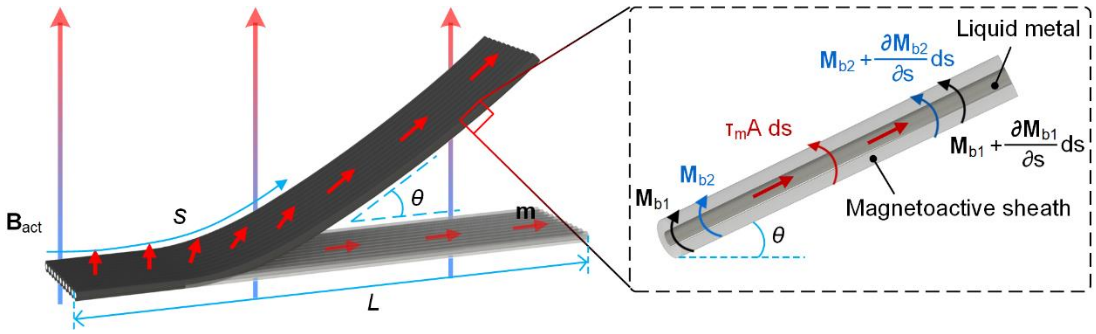

Supplementary Figure 28. Quasi-static analysis of the MME fiber. The bending moment acting on an infinitesimal section of a MME fiber under equilibrium-state deformation is shown in the inset. Therefore, On the basis of the theoretical framework developed for ferromagnetic soft materials[30], we provide the fundamental equations for quantitative description of the deformation of MME structure upon magnetic actuation. The vector **m** represents the magnetization of the infinitesimal section of the MME fiber in the initial undeformed state.

The magnetic moment $\mathbf{M}_{\mathbf{net}}$ is expressed as:

$$\mathbf{M}_{\mathbf{net}} = \int_0^s \mathbf{m}A\,\mathrm{d}s \tag{1}$$

Where A is the cross-sectional area.

Along the neutral axis of the MME structure, it can be segmented into infinitesimal coaxial elements. Deformation of each the element can be described by the Euler-Bernoulli equation of the beam, the bending moment of the sheath $M_{b1}(s) = E_1 I_1 \frac{\partial\theta(s)}{\partial s}$, bending moment of the liquid metal core $M_{b2}(s) = E_2 I_2 \frac{\partial\theta(s)}{\partial s}$.

Upon the application of an external actuating magnetic field $\mathbf{B}_{\mathrm{act}}$, deformation of the MME structure is actuated by the magnetic torque ($T_{\mathrm{m}} = M_{\mathrm{net}} \times B_{\mathrm{act}}$) The MME structure would reaches an equilibrium-state with $\mathbf{T}_{\mathbf{m}} = \mathbf{T}_{\mathbf{e}}$, where $\mathbf{T}_{\mathbf{e}}$ is the passive elastic torque from deformation.

Therefore, the equilibrium equation is established as:

$$\int_0^s T_{\mathbf{m}}(s)\mathrm{d}s = M_b(s) = M_{b1}(s) + M_{b2}(s) \tag{2}$$

where E_1 and E_2 are Young's moduli of the sheath and the liquid metal core, respectively. I_1 and I_2 are the moments of inertia of the sheath and the liquid metal core, respectively.

The curvature k(s) at position s can be calculated as:

$$k(s) = \frac{d\theta(s)}{ds} = \frac{M_{\mathrm{net}} \times B}{E_1 I_1 + E_2 I_2} \tag{3}$$

where $\theta(s)$ is the rotation angle at position s, which can describe the target shape of the MME structure programmed under the continuous magnetization profile **m**, the total rotation angle $\theta(s)$ at x is expressed as:

$$\theta(s) = \int_0^x \int_0^s \frac{m \times BA}{E_1 I_1 + E_2 I_2}\mathrm{dsds} \tag{4}$$

According to equations 3 and 4, when the magnetization profile **m** of the MME structure was given, its deformation degree mainly depended on the strength of the actuating magnetic field B_{act}.

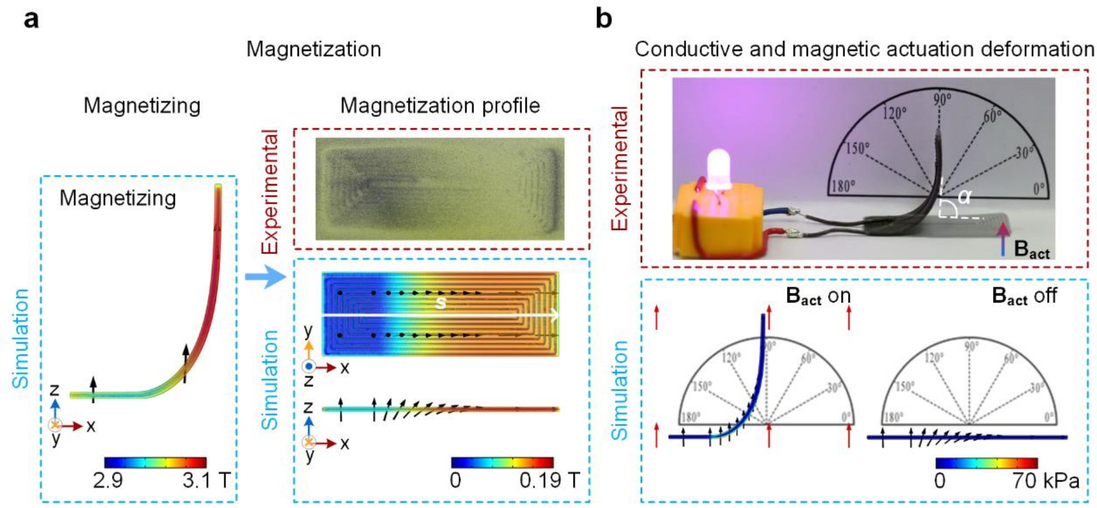

Supplementary Figure 29. Hybrid actuation and conduction capabilities for the MME coil structure. (a) The simulation and experimental results of the magnetization profile **m** of the MME coil structure. (b) The simulation and experimental results of magnetic actuation deformation of the MME coil structure. As the actuation magnetic field $\mathbf{B}_{act}$ increases, the bending angle of the MME coil structure in the flexible state increases from 0° to 90°. The bending angle of the MME structure can be precisely controlled by actuation magnetic field $\mathbf{B}_{act}$.

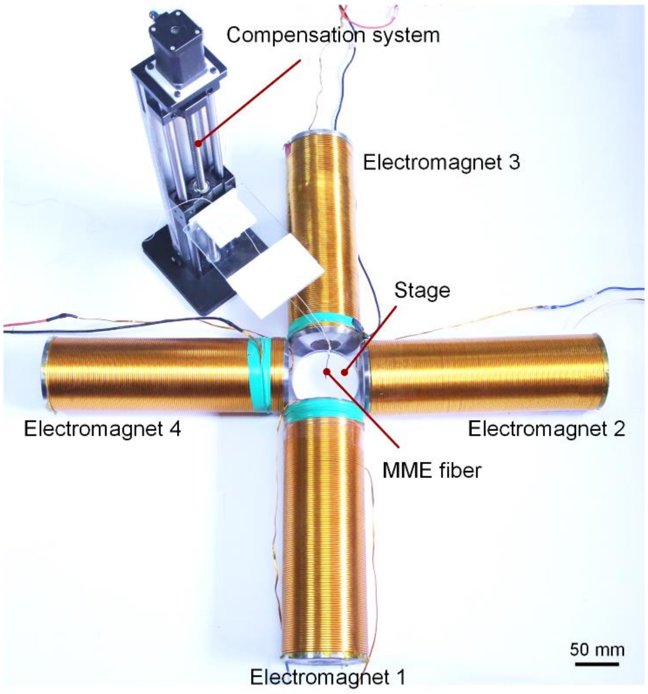

Supplementary Figure 30. The magnetic navigation system consists of a current control system, a stage, a compensation system, and four electromagnets (outer diameter: 100 mm; length: 300 mm).

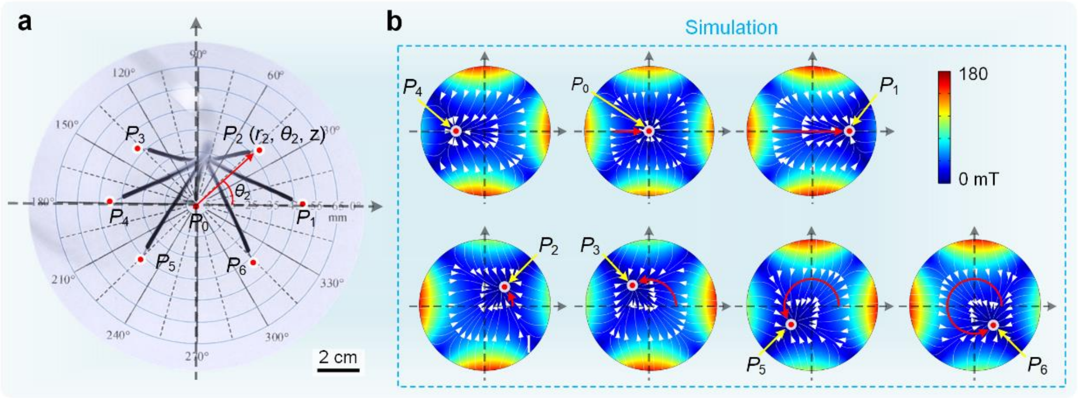

Supplementary Figure 31. Actuation of the catheter-style soft surgical tool. (a) The operating space of the catheter-style soft surgical tool is shown in the polar coordinate system P (r, θ, z). (b) The simulation results of various actuation magnetic field distributions correspond to points P_0-P_6. P_0 (0 mm, 0°, 0), P_1 (22.5 mm, 0°, 0), P_2 (17.5 mm, 45°, 0), P_3 (17.5 mm, 135°, 0), P_4 (17.5 mm, 180°, 0), P_5 (17.5 mm, 225°, 0), P_6 (17.5 mm, 315°, 0).

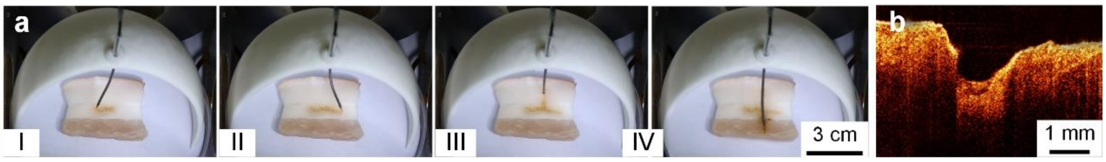

Supplementary Figure 32. The catheter-style soft surgical tool was delivered from an entrance hole for magnetically controlled ablation of a porcine tissue in the enclosed space. (a) The process of magnetically controlled ablation. (b) Optical coherence tomography (OCT) image of tissue after ablation.

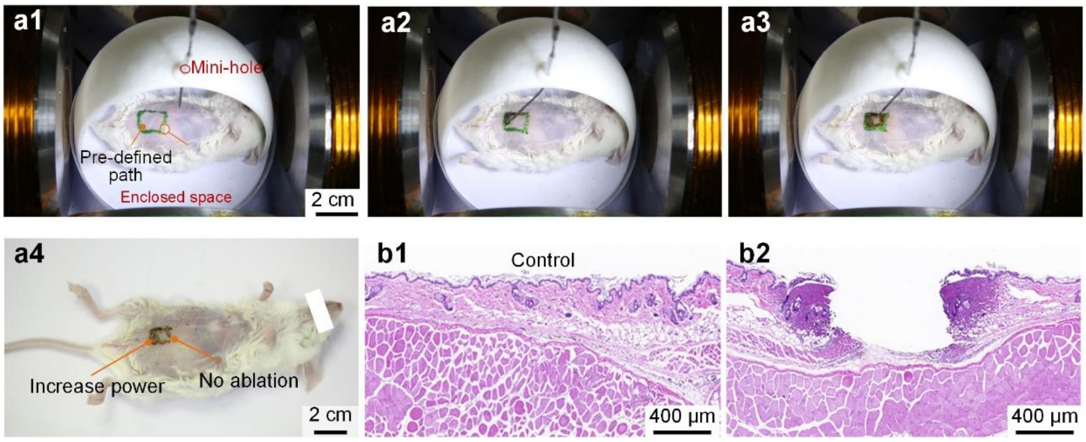

Supplementary Figure 33. *In vivo* minimally invasive electro-ablation surgery on rat skin with magnetic navigation. An anesthetized rat was placed on an operating table. Electrical ablation was performed along a pre-defined path. (a1-a3) Images showing the electro-ablation operation on rat skin; the green frame represents the pre-operatively planned ablation path. (a4) Results of ablation surgical. (b1-b2) Representative H&E staining of skin after ablation.

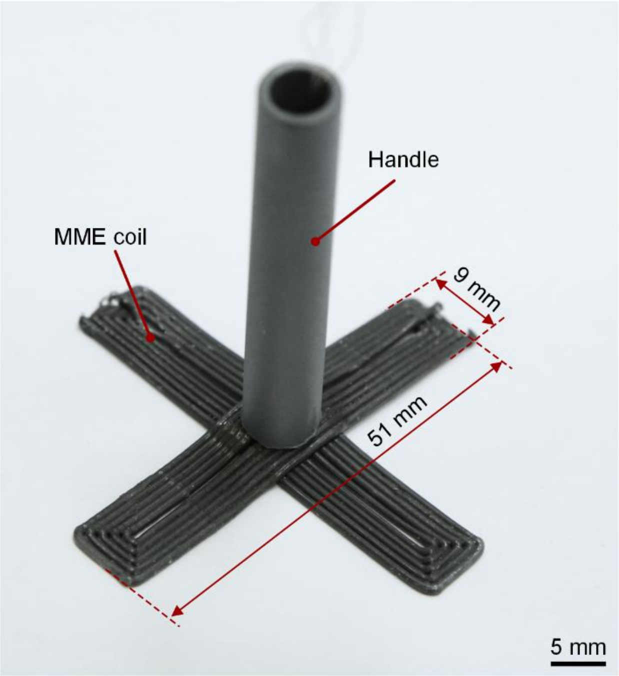

Supplementary Figure 34. The soft MME gripper printed by coaxial printing was composed of two orthogonal MME coils (51×9 mm^2) and a handle.

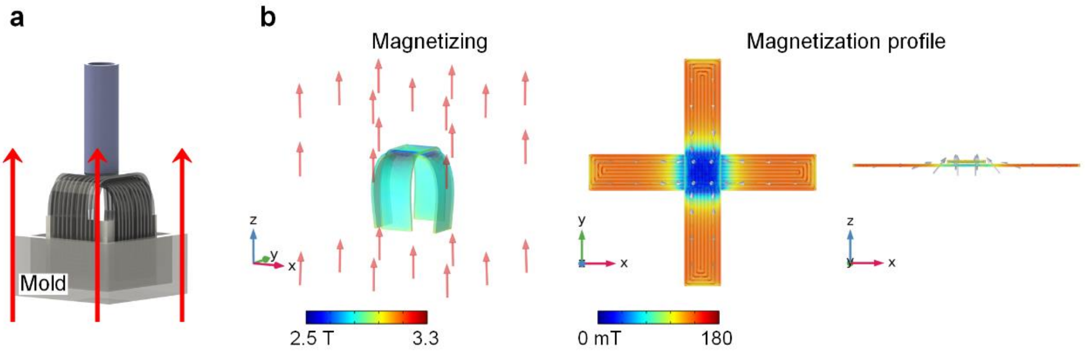

Supplementary Figure 35. Magnetization of the MME gripper. (a) Magnetization process of the MME holder with the assistance of a mold. (b) Simulation results showing the magnetizing process in the deformed state and the resulted magnetization profile **m** of the MME gripper in the deformation-free state.

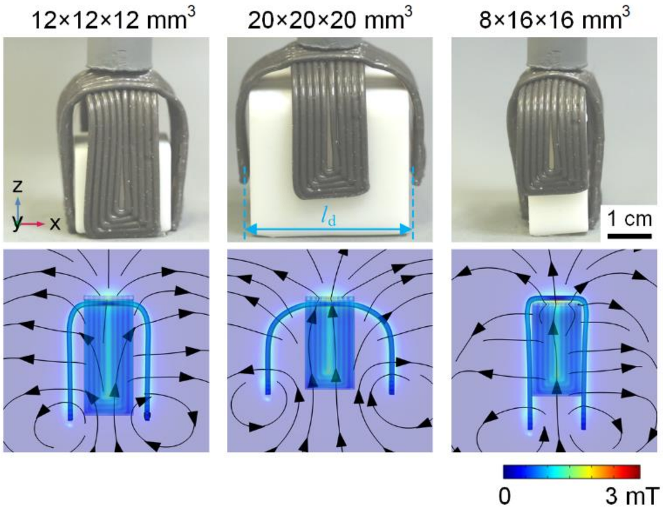

Supplementary Figure 36. Deformation of the MME gripper grasping objects of different sizes (top) and the corresponding induced magnetic field $\mathbf{B}_{\mathrm{ind}}$ from simulation (bottom). In the deformation process, the induced magnetic field $\mathbf{B}_{\mathrm{ind}}$ of the MME gripper from two halves of the coils would increasingly repeal each other as the distance (l_{d}) between the two edges of the MME coil gripper decreases.

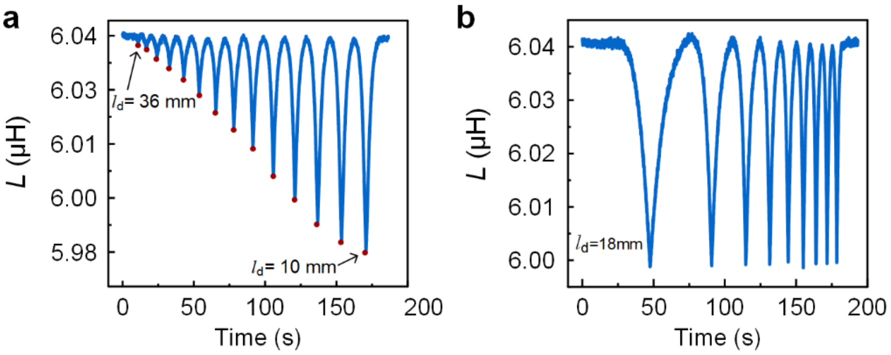

Supplementary Figure 37. Inductance variation characteristics of the MME gripper. (a) The inductance variation of the MME gripper when deformed with different spacing l_d. As l_d decreased from 36 mm to 10 mm, the L decreased from 6.037 μH to 5.981 μH. (b) The inductance of the MME gripper at different speeds at a constant l_d. The relative change in inductance L is independent of the grasping speeds from 0.5 mm/s to 4.0 mm/s, revealing that the MME gripper can stably sense the object size independent of the grasping speed.

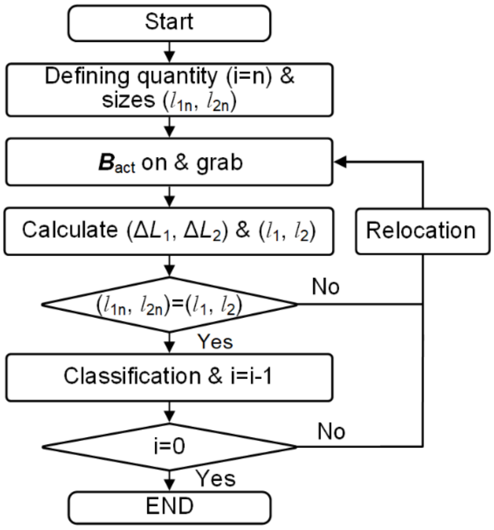

Supplementary Figure 38. Control procedures for subject identification and sorting by the MME gripper. First, define the object quantity (number = n) and the geometric dimensions (l_{1n}, l_{2n}) for each object; grab the object and measure the inductance change of the gripper (ΔL_1, ΔL_2). Based on the quantitative relationship between ΔL and l_d, calculate the size of the object for object sorting.

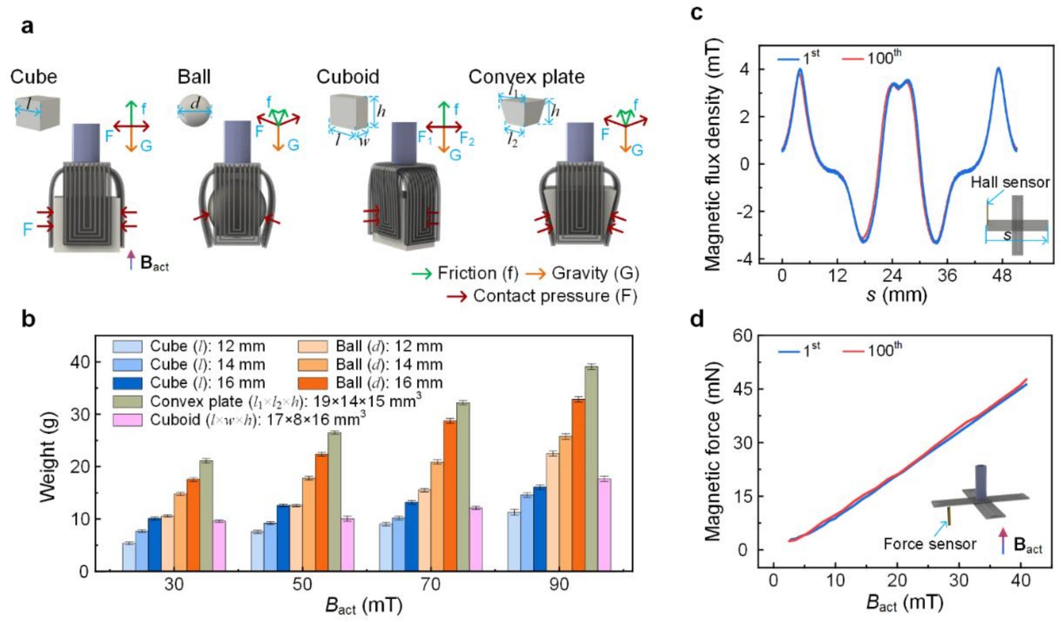

Supplementary Figure 39. Gripping capability and durability of the MME gripper. (a) Grasping objects of different shapes by the MME gripper. (b) The maximum weight for objects with different shapes that can be grasped by the MME gripper under different magnetic strength. Error bars are one standard deviation, and the number of independent experiments $n = 3$. (c) Experimental results of the magnetic field distribution for the MME gripper before and after cyclic grasping (100 cycles; cube: 12×12×12 mm^3; weight: 16.08 g). (d) The grasping force of the MME gripper before and after cyclic grasping as a function of the strength of the actuation magnetic field.

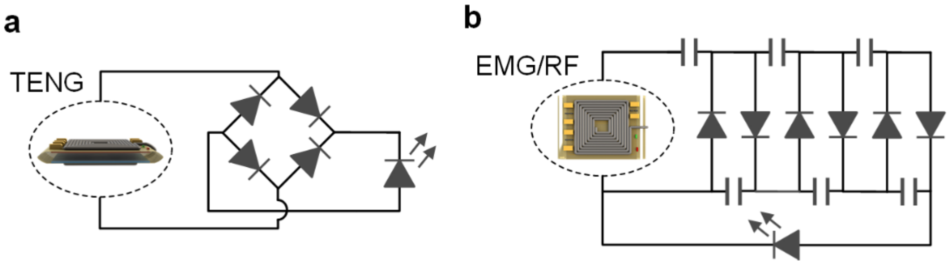

Supplementary Figure 40. Schematic diagram of the soft MME robot's circuit. (a) Schematic diagram of the rectification circuit of TENG. (b) Schematic of the boost rectifier circuit of EMG/RF.

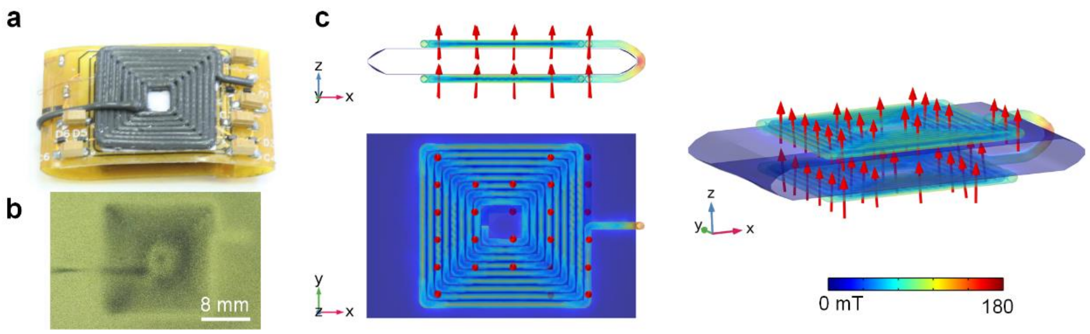

Supplementary Figure 41. The magnetization distribution of the soft MME robot. (a) The optical image of the soft MME robot prototype. The magnetization profile **m** of the soft MME robot from experiment (b), and (c) simulation.

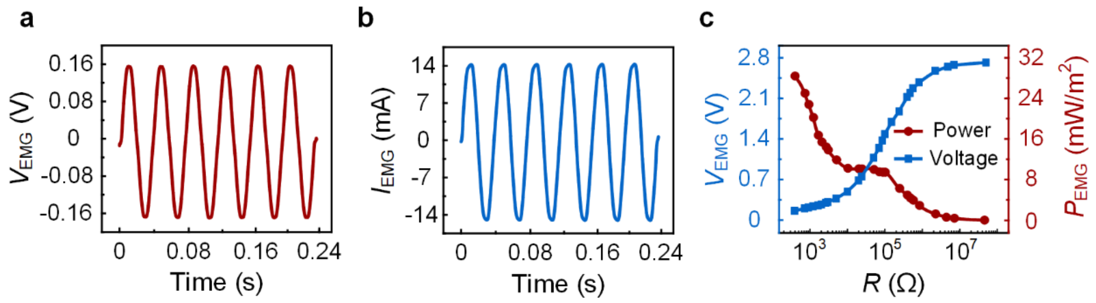

Supplementary Figure 42. The electrical output performance of the EMG, (a) voltage, (b) current, and (c) output voltage and the corresponding power. As shown in Supplementary Fig. 42a-b, at an actuation magnetic field strength of 165 mT (frequency: 24 Hz), the output open-circuit voltage (V_{EMG}) and short-circuit current (I_{EMG}) of the TENG can reach 0.16 V and 14.1 mA, respectively. Supplementary Fig. 42c shows the output characteristics of EMG after boosting by the boosting circuit (the boosting amplitude is 6 times). As the load resistance increases, the output voltage of the EMG increases to 2.71 V, and the maximum output power of the EMG (about 28.9 mW/m^2) decreases with the load resistance.

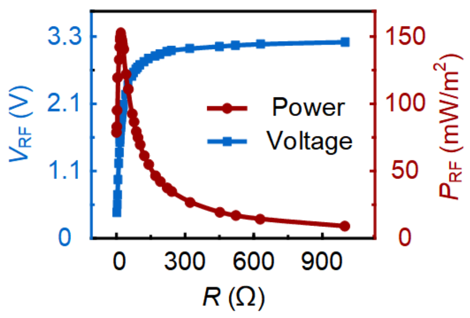

Supplementary Figure 43. Output voltage and the corresponding output power in the RF state for wireless energy transmission. Output characteristics of RF after boosting, as shown in Supplementary Fig. 43 (D_{RF} = 28 mm). With the increase of load resistance, the output voltage of RF gradually increases to 3.1 V, and the maximum output power of RF is about 163 mW/m^2 when the load resistance is about 19 Ω.

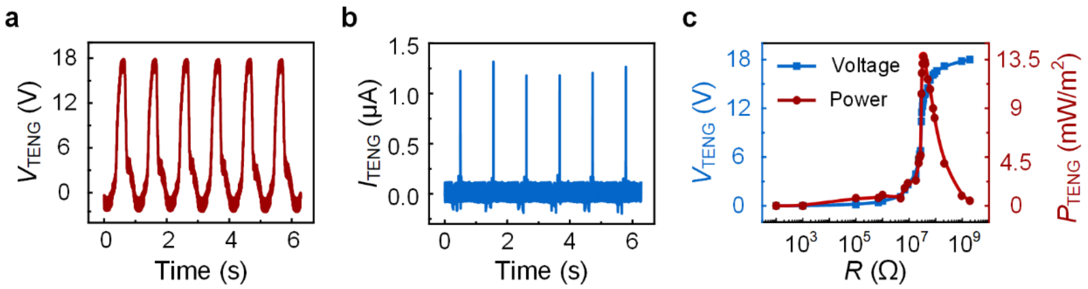

Supplementary Figure 44. The electrical output performance of the TENG, voltage signal (a), current signal (b), Output voltage and the corresponding power signals (c). TENG can produce an open-circuit peak voltage V_{TENG} of 18 V, a short-circuit peak current I_{TENG} of 1.2 μA, and a peak power density P_{TENG} of 13.8 mW/m^2 at an external resistance load of 42 MΩ (B_{act} = 180 mT, f_{act} = 1 Hz).

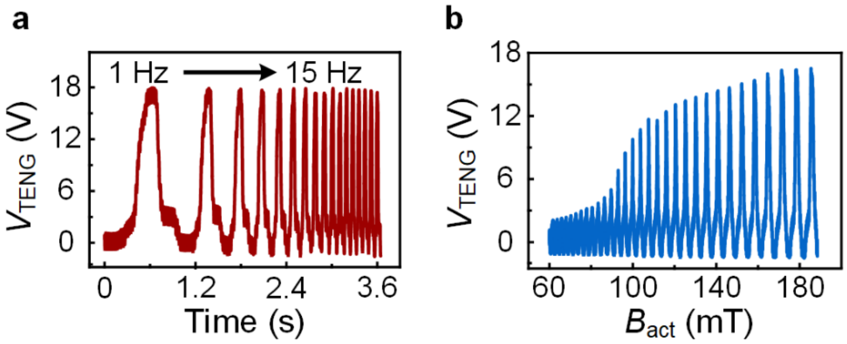

Supplementary Figure 45. The electrical output performance of TENG as a function of magnetic field frequency and strength. (a) The output voltage at different actuation magnetic field frequency from 1 Hz to 15 Hz. (b) The output voltage under different actuation magnetic field strength from 60 mT to 190 mT.

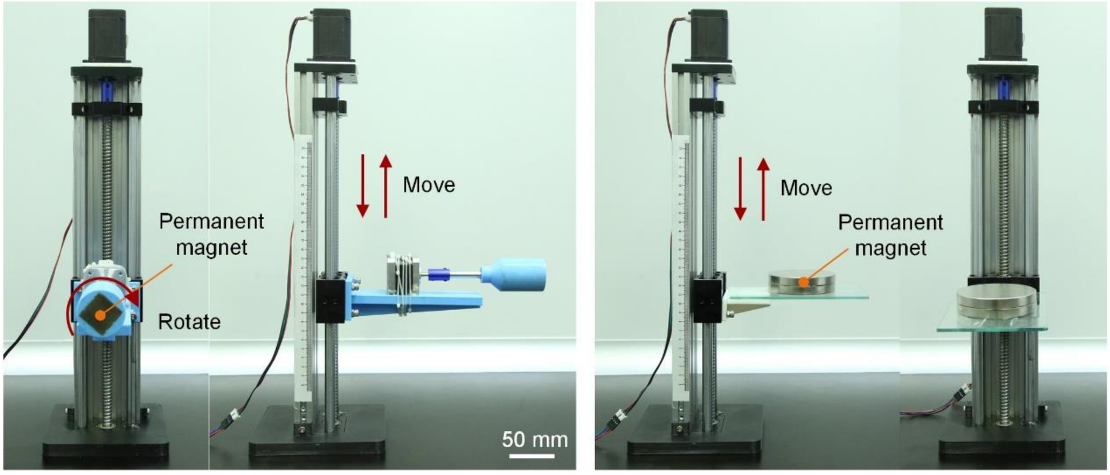

Supplementary Figure 46. Change the direction and strength of the actuation magnetic field by manipulating the position and orientation of the magnet through a custom-designed steering system.

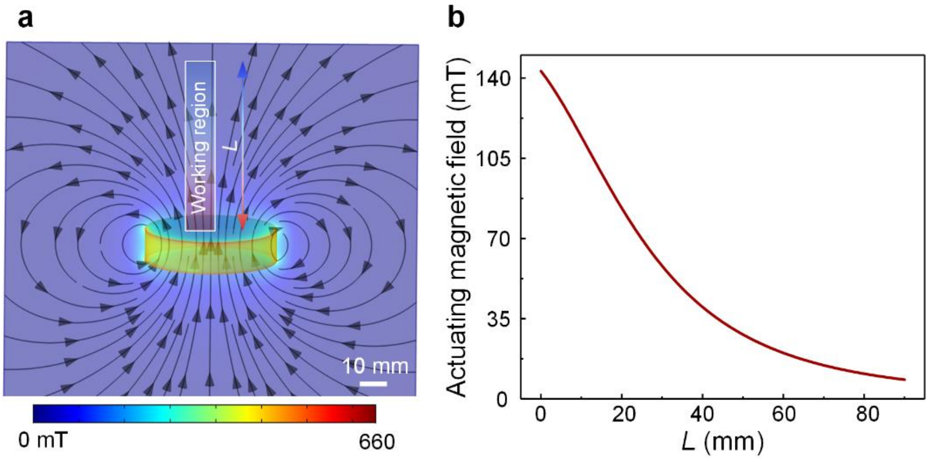

Supplementary Figure 47. The driving magnetic field generated by the permanent magnet. (a) The actuation magnetic field is generated by a cylindrical NdFeB permanent magnet. (b) Variation of actuation magnetic field strength along the center of the cylindrical permanent magnet.

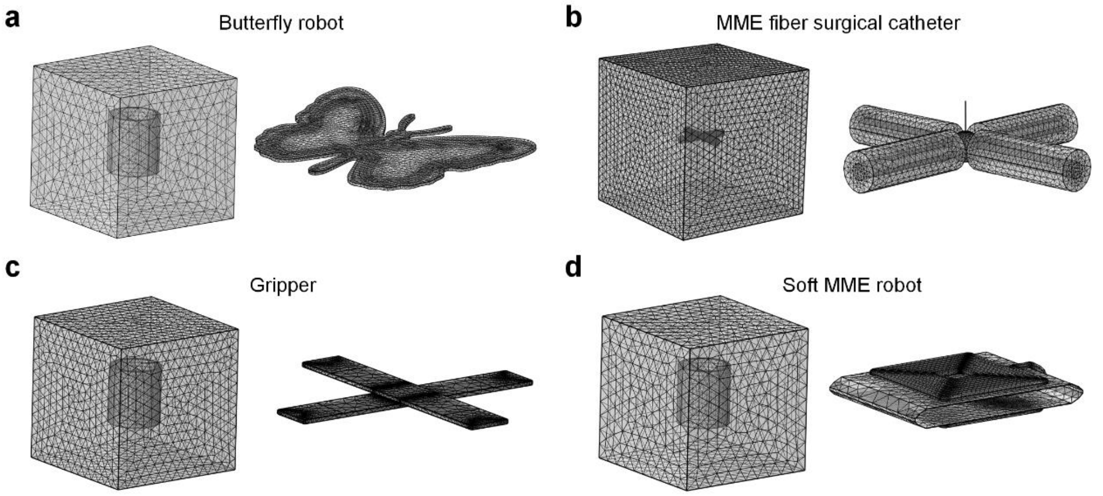

Supplementary Figure 48. FEA models of Meshed model of the MME structures used in the FEA. (a) the butterfly robot. (b) the catheter-style soft surgical tool. (c) the soft MME gripper. (d) the soft MME robot.

References

1. Zhang J., Soon R.H., Wei Z., et al. Liquid Metal-Elastomer Composites with Dual-Energy Transmission Mode for Multifunctional Miniature Untethered Magnetic Robots. *Adv. Sci.* **6**, e2203730 (2022).
2. Hao X.P., Li C.Y., Zhang C.W., et al. Self-Shaping Soft Electronics Based on Patterned Hydrogel with Stencil-Printed Liquid Metal. *Adv. Funct. Mater.* **31**, 2105481 (2021).
3. Deng B., Cheng G.J. Pulsed Laser Modulated Shock Transition from Liquid Metal Nanoparticles to Mechanically and Thermally Robust Solid-Liquid Patterns. *Adv. Mater.* **31**, e1807811 (2019).
4. Park J.E., Kang H.S., Koo M., et al. Autonomous Surface Reconciliation of a Liquid-Metal Conductor Micropatterned on a Deformable Hydrogel. *Adv. Mater.* **32**, e2002178 (2020).
5. Zhao Y., Gao S., Zhang X., et al. Fully Flexible Electromagnetic Vibration Sensors with Annular Field Confinement Origami Magnetic Membranes. *Adv. Funct. Mater.* **30**, 2001553 (2020).
6. Zhou Q., Ji B., Hu F., et al. Magnetized Microcilia Array-Based Self-Powered Electronic Skin for Micro-Scaled 3d Morphology Recognition and High-Capacity Communication. *Adv. Funct. Mater.* **32**, 2208120 (2022).
7. Ma B., Zhang J., Chen G.S., et al. Shape-Programmable Liquid Metal Fibers. *Biosens.-Basel* **13**, 28 (2022).
8. Li G., Zhang M., Liu S., et al. Three-Dimensional Flexible Electronics Using Solidified Liquid Metal with Regulated Plasticity. *Nat. Electron.* **6**, 154-163 (2023).
9. Yu X., Fan W., Liu Y., et al. A One-Step Fabricated Sheath-Core Stretchable Fiber Based on Liquid Metal with Superior Electric Conductivity for Wearable Sensors and Heaters. *Adv. Mater. Technol.* **7**, 2101618 (2022).
10. Ning C., Wei C., Sheng F., et al. Scalable One-Step Wet-Spinning of Triboelectric Fibers for Large-Area Power and Sensing Textiles. *Nano Res.* **16**, 7518–7526 (2023).
11. Zheng L., Zhu M., Wu B., et al. Conductance-Stable Liquid Metal Sheath-Core Microfibers for Stretchy Smart Fabrics and Self-Powered Sensing. *Sci. Adv.* **7**, eabg4041 (2021).
12. Khondoker M.A.H., Ostashek A., Sameoto D. Direct 3d Printing of Stretchable Circuits Via Liquid Metal Co-Extrusion within Thermoplastic Filaments. *Adv. Eng. Mater.* **21**, 1900060 (2019).
13. Zhou L.Y., Gao Q., Zhan J.F., et al. Three-Dimensional Printed Wearable Sensors with Liquid Metals for Detecting the Pose of Snakelike Soft Robots. *ACS Appl. Mater. Interfaces* **10**, 23208-23217 (2018).
14. Wang Y., Wang Z., Wang Z., et al. Multifunctional Electronic Textiles by Direct 3d Printing of Stretchable Conductive Fibers. *Adv. Electron. Mater.* **9**, 2201194 (2023).
15. Chen Y., Liu Y., Ren J., et al. Conformable Core-Shell Fiber Tactile Sensor by Continuous Tubular Deposition Modeling with Water-Based Sacrificial Coaxial Writing. *Mater. Des.* **190**, 108567 (2020).
16. Ning C., Dong K., Gao W., et al. Dual-Mode Thermal-Regulating and Self-Powered Pressure Sensing Hybrid Smart Fibers. *Chem. Eng. J.* **420**, 129650 (2021).
17. Lai Y.C., Lu H.W., Wu H.M., et al. Elastic Multifunctional Liquid–Metal Fibers for Harvesting Mechanical and Electromagnetic Energy and as Self-Powered Sensors. *Adv. Energy Mater.* **11**,

2100411 (2021).

18. Fu C., Tang W., Miao Y., et al. Large-Scalable Fabrication of Liquid Metal-Based Double Helix Core-Spun Yarns for Capacitive Sensing, Energy Harvesting, and Thermal Management. *Nano Energy* **106**, 108078 (2023).
19. Cooper C.B., Joshipura I.D., Parekh D.P., et al. Toughening Stretchable Fibers Via Serial Fracturing of a Metallic Core. *Sci. Adv.* **5**, eaat4600 (2019).
20. Yamagishi K., Zhou W., Ching T., et al. Ultra-Deformable and Tissue-Adhesive Liquid Metal Antennas with High Wireless Powering Efficiency. *Adv. Mater.* **33**, 2008062 (2021).
21. Lin R., Kim H.J., Achavananthadith S., et al. Digitally-Embroidered Liquid Metal Electronic Textiles for Wearable Wireless Systems. *Nat. Commun.* **13**, 2190 (2022).
22. Sun X., Fu J.H., Zhao H., et al. Electronic Whiskers for Velocity Sensing Based on the Liquid Metal Hysteresis Effect. *Soft Matter* **18**, 9153-9162 (2022).
23. Hong K., Choe M., Kim S., et al. An Ultrastretchable Electrical Switch Fiber with a Magnetic Liquid Metal Core for Remote Magnetic Actuation. *Polymers* **13**, 2407 (2021).
24. Zhou X., Shu J., Jin H., et al. Variable Stiffness Wires Based on Magnetorheological Liquid Metals. *Int. J. Smart Nano Mater.* **13**, 232-243 (2022).
25. Zhao Y.S., Lo C.Y., Ruan L.C., et al. Somatosensory Actuator Based on Stretchable Conductive Photothermally Responsive Hydrogel. *Sci. Rob.* **6**, eabd5483 (2021).
26. Wang X.Q., Chan K.H., Cheng Y., et al. Somatosensory, Light-Driven, Thin-Film Robots Capable of Integrated Perception and Motility. *Adv. Mater.* **32**, e2000351 (2020).
27. Jin T., Sun Z., Li L., et al. Triboelectric Nanogenerator Sensors for Soft Robotics Aiming at Digital Twin Applications. *Nat. Commun.* **11**, 5381 (2020).
28. Truby R.L., Wehner M., Grosskopf A.K., et al. Soft Somatosensitive Actuators Via Embedded 3d Printing. *Adv. Mater.* **30**, e1706383 (2018).
29. Zhao H.C., O'Brien K., Li S., et al. Optoelectronically Innervated Soft Prosthetic Hand Via Stretchable Optical Waveguides. *Sci. Rob.* **1**, eaai7529 (2016).
30. Hu W., Lum G.Z., Mastrangeli M., et al. Small-Scale Soft-Bodied Robot with Multimodal Locomotion. *Nature* **554**, 81-85 (2018).
