# Supplementary material for: Coaxially printed magnetic mechanical electrical hybrid structures with actuation and sensing functionalities
Source: Nat Commun. 2023 Jul 22;14:4428. doi: 10.1038/s41467-023-40109-z (PMC10363174; doi:10.1038/s41467-023-40109-z)
Supplement: Supplementary file 3 — Description of Additional Supplementary Files [file 41467_2023_40109_MOESM3_ESM.pdf]

Description of Additional Supplementary Files

Supplementary Movie 1: Coaxial printing the magnetic-mechanical-electrical (MME) structure process

Supplementary Movie 2: Magnetically actuated deformation and conductive function of the magnetic-mechanical - electrical (MME) fiber

Supplementary Movie 3: The fabrication process of the butterfly robot

Supplementary Movie 4: Functional demonstration of somatosensory actuation and energy harvesting

Supplementary Movie 5: The excellent electromechanical performance of magnetic-mechanical-electrical (MME) structure

Supplementary Movie 6: The durability of the magnetic-mechanical-electrical (MME) structure immersed in acid (HCl) or alkaline (NaOH) solutions

Supplementary Movie 7: Simulation and experiments of magnetization, magnetically actuated deformation, hybrid actuation and sensing in coaxially printed magnetic-mechanical-electrical (MME) structures

Supplementary Movie 8: Experiment and simulation of electroablation surgery based on the catheter-style soft surgical tool

Supplementary Movie 9: Experiment and simulation of magnetically actuated magnetic-mechanical-electrical (MME) gripper with in-situ sensing

Supplementary Movie10: Locomotion and three modes of energy generation modes of the magnetic-mechanical - electrical (MME) soft robot
