# Supplementary material for: Author Correction: Coaxially printed magnetic mechanical electrical hybrid structures with actuation and sensing functionalities
Source: Nat Commun. 2026 Oct 22;17:11035. doi: 10.1038/s41467-026-78963-2 (PMC13691099; doi:10.1038/s41467-026-78963-2)
Supplement: Supplementary file 1 [file 41467_2026_78963_MOESM1_ESM.pdf]

Original, uncorrected figures

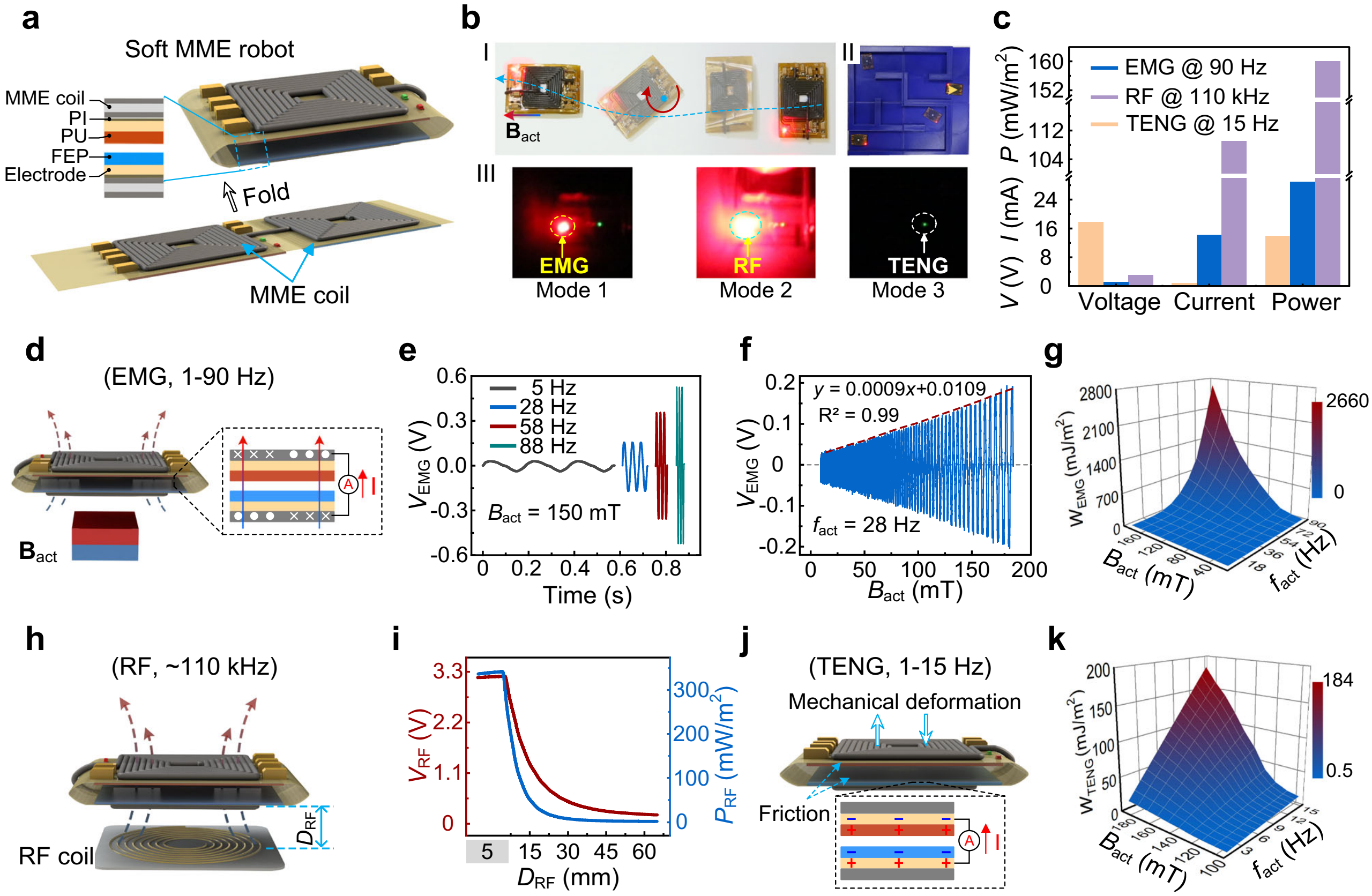

Fig. 7 | Hybrid actuation and wireless energy transmission enabled by a soft magnetic-mechanical-electrical (MME) robot.

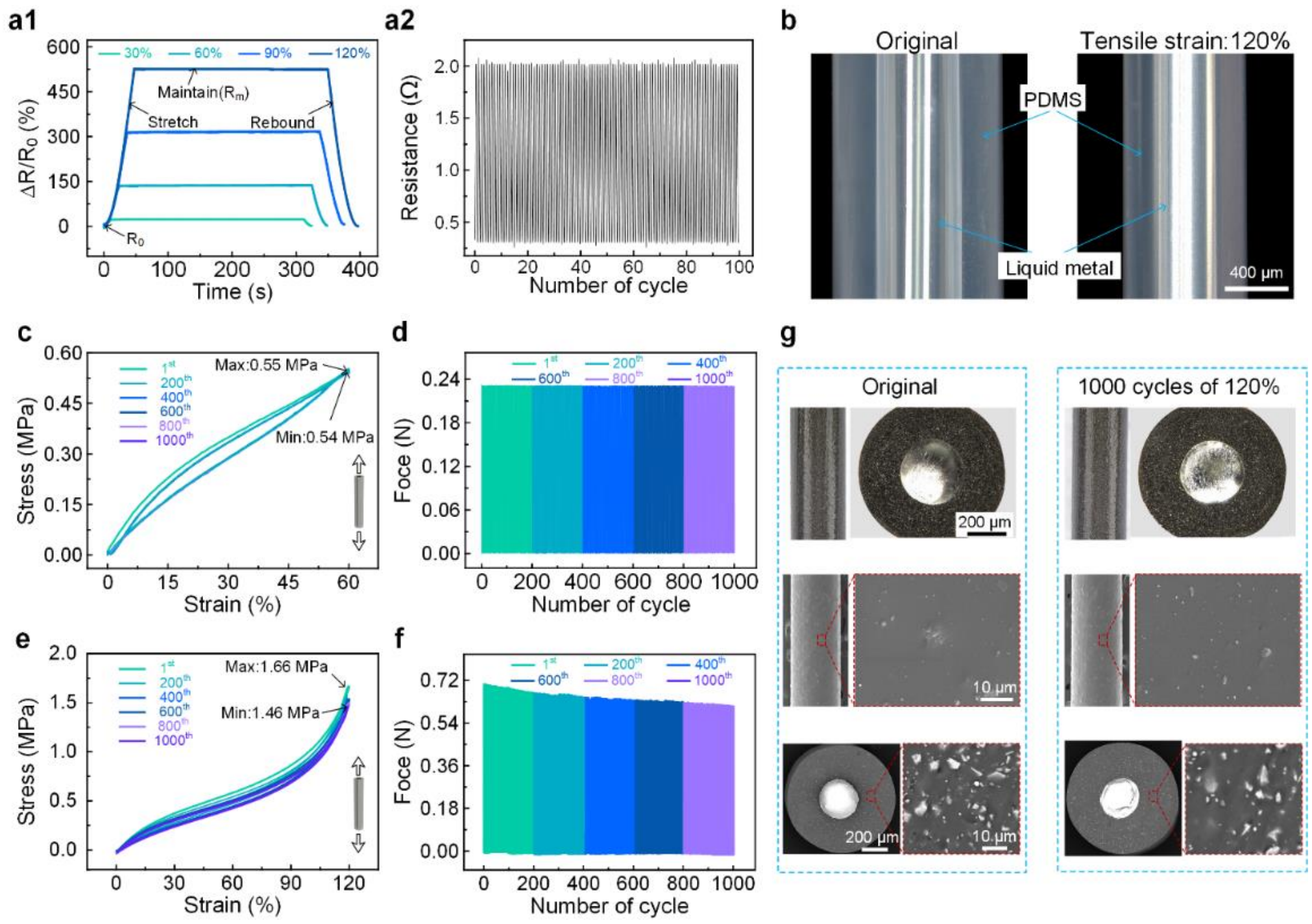

Supplementary Figure 22.
